# *Bordetella pertussis,* the Causative Agent of Whooping Cough, Evolved from a Distinct, Human-Associated Lineage of *B. bronchiseptica*

**DOI:** 10.1371/journal.ppat.0010045

**Published:** 2005-12-30

**Authors:** Dimitri A Diavatopoulos, Craig A Cummings, Leo M Schouls, Mary M Brinig, David A Relman, Frits R Mooi

**Affiliations:** 1 Laboratory for Vaccine-Preventable Diseases, National Institute of Public Health and the Environment, Bilthoven, Netherlands; 2 Eijkman Winkler Institute, University Medical Center, Utrecht, Netherlands; 3 Department of Microbiology and Immunology, Stanford University School of Medicine, Stanford, California, United States of America; 4 VA Palo Alto Health Care System, Palo Alto, California, United States of America; 5 Department of Medicine, Stanford University School of Medicine, Stanford, California, United States of America; Washington University in St. Louis, United States of America

## Abstract

*Bordetella pertussis, B. bronchiseptica, B. parapertussis_hu_,* and *B. parapertussis_ov_* are closely related respiratory pathogens that infect mammalian species. *B. pertussis* and *B. parapertussis_hu_* are exclusively human pathogens and cause whooping cough, or pertussis, a disease that has resurged despite vaccination. Although it most often infects animals, infrequently *B. bronchiseptica* is isolated from humans, and these infections are thought to be zoonotic. *B. pertussis* and *B. parapertussis_hu_* are assumed to have evolved from a *B. bronchiseptica*–like ancestor independently. To determine the phylogenetic relationships among these species, housekeeping and virulence genes were sequenced, comparative genomic hybridizations were performed using DNA microarrays, and the distribution of insertion sequence elements was determined, using a collection of 132 strains. This multifaceted approach distinguished four complexes, representing *B. pertussis, B. parapertussis_hu_,* and two distinct *B. bronchiseptica* subpopulations, designated complexes I and IV. Of the two *B. bronchiseptica* complexes, complex IV was more closely related to *B. pertussis*. Of interest, while only 32% of the complex I strains were isolated from humans, 80% of the complex IV strains were human isolates. Comparative genomic hybridization analysis identified the absence of the pertussis toxin locus and dermonecrotic toxin gene, as well as a polymorphic lipopolysaccharide biosynthesis locus, as associated with adaptation of complex IV strains to the human host. Lipopolysaccharide structural diversity among these strains was confirmed by gel electrophoresis. Thus, complex IV strains may comprise a human-associated lineage of *B. bronchiseptica* from which *B. pertussis* evolved. These findings will facilitate the study of pathogen host-adaptation. Our results shed light on the origins of the disease pertussis and suggest that the association of *B. pertussis* with humans may be more ancient than previously assumed.

## Synopsis

*Bordetella pertussis* causes whooping cough, which kills 300,000 persons annually, and is reemerging despite vaccination. This human-restricted species is closely related to the respiratory pathogens *B. parapertussis_hu_*, which is also human restricted, and *B. bronchiseptica*, which infects a broad range of mammals. Based on its limited genetic diversity and lack of historical descriptions, it has been suggested that the association between *B. pertussis* and humans is recent. In this study, the authors examined the genetic diversity and evolutionary relationships of these three *Bordetella* species. Their results suggest that *B. parapertussis* evolved from an animal-associated lineage of *B. bronchiseptica*, while *B. pertussis* evolved from a distinct *B. bronchiseptica* lineage that may already have had a preference for hominids up to 2.5 million years ago. Extant members of this newly identified *B. bronchiseptica* lineage were found to circulate in human populations. Comparisons of gene content revealed genomic features that are shared by and specific to *B. pertussis* and the *B. bronchiseptica* human-associated lineage and that may be important for association with the human host. These two lineages also have differences in key virulence genes that may reflect immune competition in the human host. By elucidating the evolutionary origins of human-adapted *Bordetella*, this study sets the stage for identification of key molecular events in host adaptation.

## Introduction

The genus *Bordetella* is composed of several species, of which three are exclusively respiratory pathogens of mammalian hosts: *B. bronchiseptica, B. pertussis,* and *B. parapertussis* (henceforth referred to as the mammalian bordetellae). *B. bronchiseptica* causes chronic and often asymptomatic respiratory tract infections in a wide variety of mammals. It is only sporadically isolated from humans [1,2], particularly from immunocompromised individuals, and human infections have been considered to be zoonotic [3]. *B. parapertussis* consists of two distinct lineages: one found in humans and the other found in sheep (*B. parapertussis_hu_* and *B. parapertussis_ov_* respectively) [4]. *B. pertussis* and *B. parapertussis_hu_* have been isolated only from humans and cause acute, transient infections and disease, designated whooping cough or pertussis. Pertussis is especially severe in young, unvaccinated children and has reemerged in recent years in vaccinated populations [5–7]. Previous research indicated that *B. pertussis* and *B. parapertussis_hu_* independently evolved from a *B. bronchiseptica*–like ancestor [8.9], and comparison of the genomes of the three isolates chosen for sequencing suggested that the time to the last common ancestor (LCA) for *B. pertussis* and *B. parapertussis_hu_* and for *B. bronchiseptica* was 0.7 to 3.5 and 0.27 to 1.4 million years, respectively [10]. Despite their different host tropisms, the mammalian bordetellae are very closely related [8,9]. Analysis of their genome sequences revealed that the adaptation of *B. pertussis* and *B. parapertussis_hu_* to the human host was accompanied by extensive genome decay [10].

Their differences in host tropism in contrast to their close genetic relationships make the mammalian bordetellae attractive candidates for the study of host-adaptation. Such studies are facilitated by the availability of genome sequences of *B. bronchiseptica*, *B. pertussis,* and *B. parapertussis_hu_* [10]. So far, only a single representative of each species has been sequenced, and it is important to determine their relationships to the *Bordetella* population as a whole. To that end and to identify genetic events that may be associated with host adaptation, we used a combination of multilocus sequence typing (MLST) [11], comparative genomic hybridization (CGH) with whole-genome microarrays [12], and the distribution of several insertion sequence elements (ISEs) to characterize 132 mammalian *Bordetella* strains with diverse host associations. This work identified two *B. bronchiseptica* lineages, the first of which is composed of mainly strains of animal origin and includes the *B. bronchiseptica* strain from which the genome sequence has been determined. The second lineage, comprising strains mainly of human origin, is more closely related to *B. pertussis* than the first lineage. Comparison of the two *B. bronchiseptica* lineages to *B. pertussis* revealed genetic differences that may be associated with adaptation to the human host.

## Results

### Population Structure of the Mammalian Bordetellae, Based on Multilocus Sequence Typing

**Figure 1:**
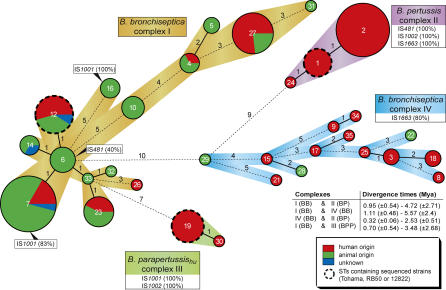
Minimum Spanning Tree of *B. bronchiseptica, B. pertussis,* and *B. parapertussis* The tree was based on the sequence of seven housekeeping genes. Individual genes were split into five subloci, and a categorical clustering was performed. In the minimum spanning tree, sequence types sharing the highest number of single locus variants were connected first. Each circle represents a sequence type (ST) the size of which is related to the number of isolates within that particular ST. Colors within circles indicate host distribution. The numbers between connected STs represent the number of different subloci between those STs. The clonal complexes (I, II, III, and IV) are indicated by colored strips between connected STs. ST16 (*B. bronchiseptica* complex I) harbors the *B. parapertussis_ov_* strains. STs containing strains of which the genome has been sequenced (*B. pertussis* Tohama, *B. parapertussis* 12822 or *B. bronchiseptica* RB50) are indicated by a thickset, dashed line. The distribution of the insertion sequence elements IS*481*, IS*1001*, IS*1002,* and IS*1663* is shown in boxes (see also Table S1); numbers between parentheses indicate the percentage of strains that contained the ISE as determined by PCR amplification. The divergence times between *B. bronchiseptica* complexes I and IV and *B. pertussis* complex II are shown.

To determine the relationships between the mammalian bordetellae, we determined the partial sequences of seven housekeeping genes from 132 strains (Table S1 and http://pubmlst.org/bordetella). We observed 32 sequence types (STs) among the 132 *Bordetella* isolates. Allele segments were divided into five equally sized subloci, and a minimum spanning tree (MST) algorithm was used to cluster the subloci [13]. Complexes were defined as groups of strains differing at fewer than five of 35 subloci with a minimum of two STs per complex. Using this criterion, strains could be assigned to one of four complexes, designated complexes I through IV (Figure 1).

Complexes II and III contained the *B. pertussis* and *B. parapertussis_hu_* isolates, respectively. Both of these complexes showed very limited genetic diversity (*H* = 0.65 and 0.35, respectively), as described previously [8,9]. *B. bronchiseptica* was divided into two distinct populations, designated complexes I and IV, respectively. The genetic diversity of these two complexes (*H* = 2.16 and 2.45, respectively) was much higher than that of complexes II and III. Complex I contained the majority of the *B. bronchiseptica* strains (76 of 91 strains), including the sequenced RB50 strain. In addition, it contained the *B. parapertussis_ov_* isolates in the study population (ST16). *B. bronchiseptica* complex IV was more closely related to *B. pertussis* than was *B. bronchiseptica* complex I. Furthermore, the host species associations of the two complexes were quite distinct. Of the *B. bronchiseptica* complex IV isolates, 80% were isolated from humans, while this was the case for only 32% of the complex I isolates. It should be noted that human *B. bronchiseptica* isolates were overrepresented in our strain collection, in comparison with their occurrence in natural populations. However, the human complex IV isolates originated from different continents, comprising North America, South America, and Europe. Previously, phylogenetic analysis based on CGH suggested the existence of a distinct *B. bronchiseptica* lineage that was closely related to *B. pertussis* [12].

**Figure 2:**
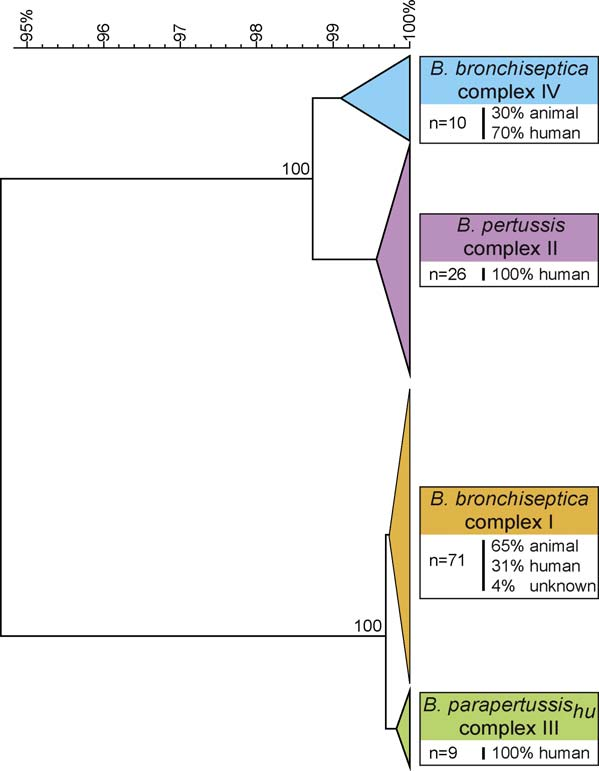
UPGMA Tree Based on the Analysis of the Pertactin Gene of *Bordetella* Isolates Used in the MLST Analysis The DNA segment coding for the extracellular domain of pertactin (P.69) was used for analysis, with the exclusion of the repeat regions 1 and 2. Bootstrap values are shown for the nodes separating the complexes and are based on 500 bootstrap replicates. The scale indicates the genetic distance along the branches. Colors of the branches indicate the four complexes defined by MLST. The number of strains of each branch is shown in boxes, as well as the host distribution.

The relationships among the mammalian bordetellae inferred from housekeeping gene sequences were confirmed by an analysis based on the pertactin gene *(prn),* which codes for a surface-associated virulence factor involved in adherence [14,15]. A UPGMA tree was constructed from the aligned *prn* sequence data, and the topology of this tree was very similar to the MLST tree (Figure 2). *B. bronchiseptica* strains are grouped into two lineages, corresponding to complex I and IV in the MLST tree. Also, *B. bronchiseptica* complex IV and *B. pertussis* strains clustered together in one branch, which was supported by bootstrapping. *B. parapertussis_hu_* comprised a separate branch within a larger cluster that also contained the *B. bronchiseptica* complex I strains. The *B. parapertussis_ov_* strains were indistinguishable from *B. bronchiseptica* complex I strains, as was observed in the MLST tree.

### Distribution of Insertion Sequence Elements

The distribution of ISEs has been used to reveal evolutionary relationships among the *Bordetella* population [9]. Toward this end, we screened our strain collection for the presence of IS*481*, IS*1001*, IS*1002*, and IS*1663* using PCR (Table S1). The distribution of the ISEs was mapped onto the MST (see Figure 1). IS*481* was detected in all *B. pertussis* strains but not in any other species, with the exception of two *B. bronchiseptica* isolates, both from a horse (B1975, B0230, ST6), consistent with previous observations [9,16]. IS*1001* was detected in all *B. parapertussis_hu_* and *B. parapertussis_ov_* strains. Additionally, IS*1001* was detected in most (21 of 25) *B. bronchiseptica* strains belonging to ST7 in complex I, but not in other STs, including STs in complex II and IV. IS*1002* was detected only in *B. pertussis* and in *B. parapertussis_hu_* strains, confirming previous observations [4,9]*.* IS*1663* [10] was detected in all *B. pertussis* isolates but also in ten of 13 *B. bronchiseptica* complex IV strains. The three complex IV strains in which IS*1663* was not detected belonged to STs 18 and 21.

### Divergence Times of Complexes

Under the assumption that the mutation rate in prokaryotes is relatively constant, the time since descent from the LCA can be estimated using pairwise mean allele distances (*K*_S_) [17,18]. The clock rates described by Whittam [19] and by Guttman and Dykhuizen [20] were used to estimate a range of divergence times between complexes. Calculations indicated that *B. pertussis* and *B. bronchiseptica* complex IV separated approximately 0.3 to 2.5 million years ago (Mya), which suggests a more recent divergence time than *B. pertussis* and *B. bronchiseptica* complex I, estimated at 1.1 to 5.6 Mya. *B. parapertussis_hu_* and *B. bronchiseptica* complex I diverged between 0.7 and 3.5 Mya according to our calculations. The divergence times of combinations of complexes is shown in Figure 1.

### Gene Content of Strains from *B. bronchiseptica* Complexes I and IV

The high percentage of complex IV strains of human origin compared to the percentage of those in complex I suggested a preference for the human host in complex IV strains. To identify genetic events that may have played a role in host adaptation or host restriction, we used CGH with *Bordetella* DNA microarrays [17,18].

Genomic DNA from 26 *B. bronchiseptica* complex I and 13 complex IV strains was hybridized to microarrays (CGH data files have been deposited in ArrayExpress, accession E-TABM-32). Significance Analysis of Microarrays (SAM) was used to identify probes with statistically significant log intensity ratio differences between the two complexes (Table S2). This approach detected sequences that have been deleted more often in one of the two complexes, as well as DNA sequences diverging from the reference sequences in one of the two complexes.

**Figure 3:**
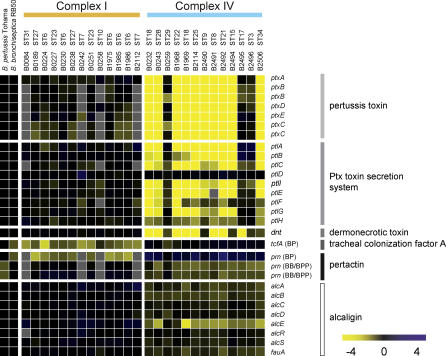
Gene Content of the Differentially Hybridizing Virulence Loci between *B. bronchiseptica* Complex I and IV, as Determined by CGH Each column represents one strain. Strain numbers and STs are indicated above the columns. Each row represents one ORF (in *B. bronchiseptica* RB50 gene order), ORF designations are shown to the right of the rows. In the case of *tcfA* and *prn*, the origins of the probes are indicated between parentheses. The BP probe of *tcfA* was 100% similar to *B. pertussis* Tohama and 85.1% similar to *B. bronchiseptica* RB50. The BP *prn* probe was 100% similar to *B. pertussis* Tohama and 86% similar to *B. parapertussis* 12822 and *B. bronchiseptica* RB50. The BB/BPP *prn* probes were both 100% similar to *B. parapertussis* 12822 (BPP) and *B. bronchiseptica* RB50 (BB) and 86% similar to *B. pertussis* Tohama. The yellow-black-blue color scale indicates the hybridization value relative to the reference; references are *B. bronchiseptica* RB50, *B. parapertussis* 12822, and *B. pertussis* Tohama. For *B. bronchiseptica* RB50 and *B. pertussis* Tohama, the data in the figure are based on the genomic sequences. Yellow indicates decreased hybridization, black indicates hybridization values comparative to the references, and blue indicates gene duplications. Intermediate values indicate partial deletions or sequence divergence. Missing data are represented in gray.

Thirty-one probes, representing 29 genes and an ISE, hybridized more strongly or more frequently to the genomes of complex IV strains than to those of complex I. Two of these probes represented the *B. pertussis* homologs of the virulence genes *prn* and *tcfA,* encoding pertactin and tracheal colonization factor, respectively [15,21] (Figure 3). Sequence analysis confirmed that the *B. bronchiseptica* complex IV *prn* sequences were more similar to those of *B. pertussis* than to those of the *B. bronchiseptica* complex I (see Figure 2).

Sixteen of these 31 probes had been identified previously as *B. pertussis*–specific [12]. In most cases, these probes hybridized to nine or more complex IV strains but not to any complex I or III strain. The genes represented by these probes encode diverse functions involved in metabolism, transport, regulation, and transposition (Table S2). With the possible exception of BP0703, which encodes a TonB-dependent iron receptor, no obvious virulence genes were observed among them. Most of the 16 genes were located in small clusters along the *B. pertussis* Tohama chromosome. The presence of these genes in *B. bronchiseptica* complex IV and *B. pertussis* but not in *B. bronchiseptica* complex I or *B. parapertussis_hu_* suggests that they were acquired by the common ancestor of *B. pertussis* and *B. bronchiseptica* complex IV.

**Figure 4:**
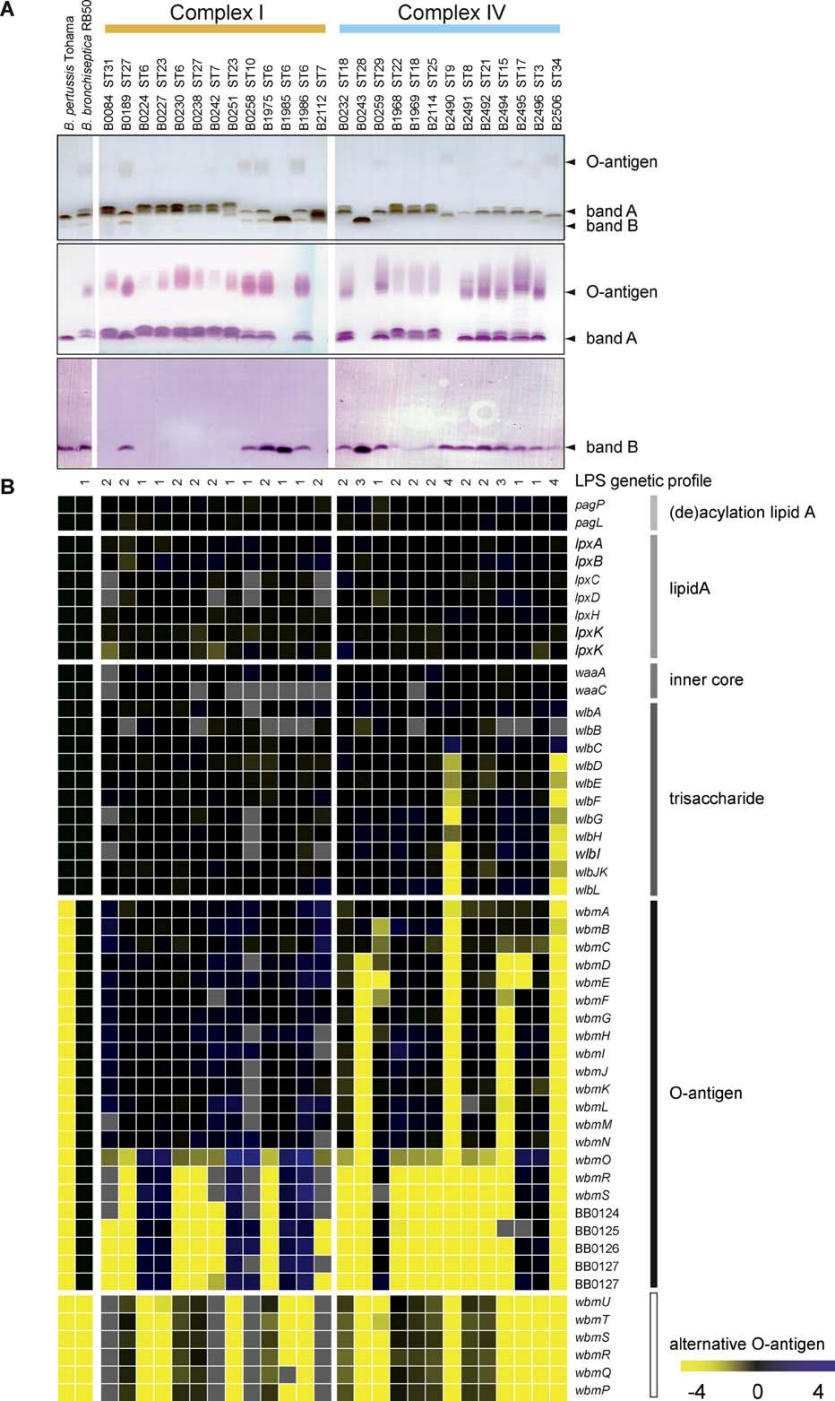
Expression of LPS by *B. bronchiseptica* Complex I and Complex IV Strains and Gene Content Variation at the LPS Biosynthesis Locus (A) Top: Electrophoretic LPS profiles obtained by tricine-SDS-PAGE and silver staining. Middle: Western blot of the same samples with mAb 36G3, which detects band A. Bottom: Western blot of the same samples with mAb BL8, which detects band B. (B) Gene content of the LPS biosynthesis locus as determined by CGH. See Figure 3 for details. For *B. bronchiseptica* RB50 and *B. pertussis* Tohama, the data in the figure are based on the genomic sequences. The genes *wbmPQRSTU* represent an alternative LPS O-antigen biosynthesis sublocus that is orthologous to the genes found in *B. parapertussis* 12822 [10] and *B. bronchiseptica* C7635E [26]. LPS genetic profiles as described in the text are indicated at the top of the columns. Color scale as in Figure 3. Missing data are represented in gray.

We also identified 248 probes, representing 237 genes, that exhibited significantly stronger hybridization to complex I genomes than to complex IV genomes (Table S2). Sixty-eight (27%) corresponded to genes associated with mobile elements such as prophages, while many of the other probes represented genes involved in metabolic, transport, and regulatory functions. Surprisingly, several virulence-associated genes were found to be missing or divergent in the complex IV strains, as compared to complex I strains. These included the *B. bronchiseptica* homologs of *tcfA* and *prn,* Bvg-regulated intermediate phase gene A *(bipA),* the alcaligin biosynthesis locus *(alcA/E),* the pertussis toxin synthesis and transport locus *(ptx/ptl),* the dermonecrotic toxin gene *(dnt),* and the lipopolysaccharide (LPS) biosynthetic locus (Figures 3 and 4).

Interestingly, ten of 13 complex IV strains harbored deletions in the *ptx* and *ptl* loci, which encode pertussis toxin (Ptx) and its secretion machinery, respectively (Figure 3) [22,23]. While conditions under which Ptx is expressed by either *B. parapertussis* or *B. bronchiseptica* have not been found, the structural genes are generally conserved among these species [24], suggesting selective pressure to retain the ability to produce functional Ptx under certain circumstances. The complex IV strains in which *ptx/ptl* was still present (ST3/17/29) were tested for expression of Ptx by immunoblotting, and these strains were found not to express Ptx under the growth conditions used (unpublished data). Another distinguishing characteristic of the complex IV strains was the deletion of the dermonecrotic toxin gene *(dnt)* in 8 of 13 strains. In contrast, this gene was detected in all *B. pertussis, B. parapertussis_hu_, B. parapertussis_ov_,* and *B. bronchiseptica* complex I strains.

### LPS Genetic and Structural Diversity in *B. bronchiseptica*

The genetic structure of the LPS biosynthesis locus differed between complex I and IV strains. The LPS molecules of Gram-negative bacteria usually consist of three, covalently linked, major domains: the lipid A, the branched chain oligosaccharide core, and the hydrophilic O-antigen. A number of genetic loci have been implicated in the synthesis of these domains in *Bordetella,* such as the *lpx* locus (lipid A), the *waa* locus (inner core), the *wlb* locus (outer core), and the *wbm* locus (O-antigen) [25–29].

*B. pertussis* LPS usually consists of lipid A and an inner core, to which the outer core (a trisaccharide) is attached; this is also referred to as band A. Certain *B. pertussis* strains produce only band B, which is identical to band A except that it lacks the trisaccharide [30]. The O-antigen, which is added to the trisaccharide, is found only in *B. bronchiseptica* and *B. parapertussis.* This structure is missing in *B. pertussis* due to the deletion of the genes *wbmA-U* [26]. In Figure 4, the gene content of the LPS locus is shown for 13 complex I and 13 complex IV strains and for *B. pertussis* Tohama and *B. bronchiseptica* RB50. Four LPS gene content profiles, designated LPS 1–4, could be distinguished among the *B. bronchiseptica* strains. Strains with the LPS 1 profile had an LPS gene composition similar to RB50, characterized by the absence of *wbmPQRSTU*. The LPS 2 profile was characterized by the absence of the genes *wbmORS* and BB0124–BB0127 and the presence of an alternative O-antigen locus, comprising *wbmPQRSTU,* orthologous to the *B. parapertussis* 12822 genes [10]. Both of these genotypes appear competent for the production of a full length LPS. Strains with the LPS 3 profile lacked *wbmD-U* and BB0124-BB0127, suggesting that they may not produce O-antigen. The LPS 4 profile was similar to the LPS 3 profile but additionally lacked *wbmABC* and *wlbD-L*, suggesting that strains of this genotype may be deficient for the production of trisaccharide as well as O-antigen. The deletion in the O-antigen genes of LPS 4 strains was similar to that observed in the O-antigen genes of *B. pertussis* Tohama. All complex I strains displayed either an LPS 1 or an LPS 2 profile. Nine of 13 complex IV strains had either an LPS 1 or an LPS 2 profile, while four complex IV strains showed more extensive deletions, resulting in LPS 3 and LPS 4 profiles.

To study the effect of these deletions on LPS production, proteinase K–treated cell lysates were analyzed by Tricine-SDS-PAGE, followed by silver staining or immunoblotting with monoclonal antibodies (mAbs) directed against either band A or band B (mAbs 36G3 and BL-8, respectively [31,32]) (Figure 4). Silver-staining showed that all complex I strains produced band A, except for B1985, which produced band B, and B2112, which produced a band migrating at a position between bands A and B. These strains showed no obvious deletions at their *wlb* or *wbm* loci, and the fact that they did not produce band A and O-antigen may be attributed to point mutations, e.g., in their *wlb* locus, or to regulatory effects. These results were confirmed by immunoblotting with mAb 36G3. The epitope in the trisaccharide that is recognized by this mAb was also present in the O-antigenic repeats, as was described previously [33]. In general, most strains that produced band A also produced an additional band just above band A. This extra band was also recognized by 36G3 and therefore is likely derived from band A.

The nine complex IV strains with LPS 1 or 2 profiles all produced band A. The two strains with the LPS 4 profile, B2490 and B2506, produced a band smaller than band A, which failed to be recognized by mAb 36G3. Of the two strains with an LPS 3 profile, one strain (B0243) produced only band B and no O-antigen. Unexpectedly, the other strain, B2494, produced band A and O-antigen as detected by immunoblotting, indicating that this strain contains as yet uncharacterized O-antigen biosynthesis genes. Silver staining also suggested that the LPS 4 strains may produce O-antigen, although the O-antigen failed to be recognized by mAb 36G3.

## Discussion

Although it has long been speculated that *B. pertussis* evolved from a *B. bronchiseptica* strain [8,9], a specific lineage has not been identified. Here we identify and characterize such a *B. bronchiseptica* lineage. Analysis of MLST data from the mammalian bordetellae identified four distinct complexes. Complex I and IV comprised *B. bronchiseptica* strains, while complex II and III comprised the human pathogens *B. pertussis* and *B. parapertussis_hu_*, respectively. Our results suggest that *B. pertussis* and *B. parapertussis_hu_* evolved from complexes I and IV, respectively, indicating that adaptation to humans occurred as two independent events, consistent with previous data [9,10].

The population structure of the mammalian bordetellae inferred from MLST data largely corresponded with a maximum parsimony phylogeny derived from a previous CGH study [12], with the exception of the relationship of *B. parapertussis_ov_* and *B. parapertussis_hu_*. In the current study, *B. parapertussis_hu_* and *B. parapertussis_ov_* are clearly derived from different STs in complex I. Further, in contrast to *B. parapertussis_hu_*, *B. parapertussis_ov_* is actually part of *B. bronchiseptica* complex I. The closer relationship between the sheep- and human-derived *B. parapertussis* lineages that was inferred from CGH data may be an artifact of long-branch attraction in the maximum parsimony tree [34].

Consistent with previous studies [8,9,35], *B. pertussis* and *B. parapertussis_hu_* showed a relatively low degree of genetic diversity, suggesting that they evolved recently or encountered a recent evolutionary bottleneck. Of the three *B. pertussis* STs observed, two were found exclusively before 1960, whereas all modern strains belong to ST2. The temporal shift in *B. pertussis* STs is consistent with our previous studies on antigenic shifts that show major changes in the *B. pertussis* population after the introduction of mass vaccination against pertussis in the 1950s and 1960s [35,36].

Most human disease is by far caused by *B. pertussis,* and we therefore focused on the relationship of *B. bronchiseptica* complex IV with *B. pertussis. B. bronchiseptica* complex IV strains were found to be more closely related to *B. pertussis* than to the complex I *B. bronchiseptica* strains. A tree based on *prn* nucleotide sequences also suggested a closer relationship of complex IV strains to *B. pertussis* than to complex I strains. A number of other features of complex IV strains were consistent with their close relationship with *B. pertussis*. Most complex IV strains were isolated from humans (80%), while the majority of complex I strains were of animal origin (68%). Almost all *B. bronchiseptica* complex IV strains were isolated from patients with whooping cough symptoms. Further, complex IV strains and *B. pertussis* shared an IS element, IS*1663,* that was not found outside these two lineages. The sharing of an IS element may be explained by either vertical or horizontal transfer. The former suggests a common ancestry, while the latter would point to niche sharing of *B. pertussis* and *B. bronchiseptica* complex IV. It seems unlikely that the association of complex IV strains with humans is due to a sampling artifact, as the strains analyzed were from widely separated geographic regions, including North America, South America, and Europe. Thus, these strains were not epidemiologically related.

Three STs (ST12, ST23, and ST27) found in complex I also contained a high percentage of human strains (55%, 43%, and 77%, respectively). All other, nonhuman isolates of these STs were collected from domesticated animals. Thus, both complex I and IV contain *B. bronchiseptica* strains that are well adapted to the human host. However, the particular relevance of the human-associated lineage in complex IV appears to be its evolutionary relationship with *B. pertussis*. The high frequency of human isolates observed in complex IV may be due to the close interaction of humans with the animal hosts in which these strains reside or to the fact that complex IV strains are better adapted to a human environment than *B. bronchiseptica* complex I strains. In either case, the *B. bronchiseptica* complex IV infections of humans would be zoonotic. Another intriguing possibility is that *B. bronchiseptica* complex IV strains are to a large extent adapted to the human host and primarily transmitted between humans.

Microarray-based CGH revealed 29 genes and IS*1663* to be more frequently present in, or more similar to, *B. pertussis* orthologs in *B. bronchiseptica* complex IV than to complex I strains. Of these genes, 16 were unique to *B. pertussis* and *B. bronchiseptica* complex IV, suggesting they were acquired after the common ancestor of complex IV and *B. pertussis* diverged from complex I. With the exception of *prn* and *tcfA,* which hybridized more strongly to the *B. pertussis*–derived probe, no known virulence genes were identified among these 30 genes. Conversely, 237 genes were absent or divergent in complex IV compared to complex I strains, suggesting that the *B. bronchiseptica* complex IV genome is decaying, as has been assumed for *B. pertussis* and *B. parapertussis_hu_*. Genome decay has been associated with host restriction or niche change in a number of pathogens such as *Yersinia pestis* [37,38] and *Burkholderia mallei* [39] and has been suggested to be a driving force of host restriction for *B. pertussis* and *B. parapertussis* as well [10]. Likewise, the apparent preference of complex IV strains for humans may also be associated with genome decay. Because putative complex IV–specific sequences were not represented on the microarray used here, we were unable to address the possibility that complex IV strains have acquired, through lateral transfer, genetic loci that may have promoted host preference. However, gene acquisition appears to have been a rare event in the evolution of *B. pertussis* and *B. parapertussis* from *B. bronchiseptica* complex I [10].

Differences between complex IV strains and *B. pertussis* were observed with respect to three major virulence factors, Ptx, Dnt, and LPS. All *B. bronchiseptica* complex I strains examined contained intact Ptx genes. Although conditions under which the Ptx genes are expressed in *B. bronchiseptica* have not been identified, their conservation suggests that they may confer a selective advantage in the ecology of complex I strains. In contrast to complex I strains, the genes encoding Ptx and its secretion apparatus were deleted from most complex IV strains (10 of 13). In the strains that did retain the Ptx locus, no in vitro expression of Ptx was observed, even though these strains were closely related to *B. pertussis*.

Another characteristic that sets complex IV strains apart from all other mammalian bordetellae is that in eight of 13 strains, *dnt* was deleted. Dnt is an intracellular toxin that activates the small GTPase Rho through deamidation or polyamination [40]. It has been shown that Dnt is important for turbinate atrophy and the colonization of the upper respiratory tract by *B. bronchiseptica* in pigs [41].

Based on CGH, four LPS genetic profiles were distinguished. The LPS genetic locus was generally more polymorphic in complex IV strains than in complex I strains, and deletions were observed in the O-antigen and trisaccharide biosynthesis genes in some complex IV strains. In complex IV strains with the LPS 4 profile, the extent of deletion in the O-antigen genes was very similar to that seen in *B. pertussis* Tohama. *B. pertussis* does not produce repetitive O-antigen as it lacks the *wbm* genes but makes a lipo-oligosaccharide that consists of lipid A to which a single trisaccharide is attached [26]. Like *B. pertussis,* four complex IV strains lacked the O-antigen genes known to be present in the sequenced genomes of *B. bronchiseptica* and *B. parapertussis_hu_*. Despite the absence of these genes, in at least one of these strains O-antigen was detected by immunoblotting, suggesting that this strain carries LPS genes distinct from those in RB50 or 12822. The two LPS 4 strains, both isolated from humans, also lacked the genes required for biosynthesis of the trisaccharide and failed to produce trisaccharide detectable by immunoblotting. The absence of the trisaccharide is intriguing in view of the fact that it was found to be otherwise conserved in all *Bordetella* strains analyzed.

It seems likely that in addition to gene loss and acquisition, differences in gene regulation have significantly contributed to host adaptation [42]. The differences observed between complex IV strains and *B. pertussis*, particularly with respect to Ptx, Dnt, and LPS, may be due to differences in niches occupied. Another possibility is that these differences have arisen in response to immune competition between *B. bronchiseptica* complex IV strains and *B. pertussis*. Gupta and co-workers [43] provided evidence that immunodominant surface antigens are organized into nonoverlapping combinations as a result of selection by the host immune system. This process could also have driven the inactivation and conservation of virulence factors in mammalian bordetellae infecting the same host. In a similar vein, Bjørnstad and Harvill [44] hypothesized that, since *B. pertussis* and *B. parapertussis_hu_* both infect humans, they may have evolved to evade cross-immunity by the other pathogen. The authors propose that immune competition provides an explanation for differences observed between *B. pertussis* and *B. parapertussis_hu_*. For example, *B. pertussis* but not *B. parapertussis_hu_* expresses Ptx, although both contain the required genes. Conversely, *B. parapertussis_hu_* expresses O-antigen, while the corresponding genes have been deleted from *B. pertussis*. Similarly, the deletion of the genes for Ptx, Dnt, and genes involved in trisaccharide syntheses by complex IV strains may have been driven by immune competition with *B. pertussis* and possibly also with *B. parapertussis_hu_*_._

The origin of the disease whooping cough is still a mystery. Although the disease has very typical symptoms in children and was one of the major causes of child mortality before the introduction of vaccination, the first written reference to the disease in Europe is found in 1540 [45]. The first description of an epidemic, which occurred in Paris, was given by Baillon in 1578 [46]. Particularly interesting are the observations made by Nils Rosen von Rosenstein in 1766, who wrote [47], “The hooping cough never appeared in Europe originally, but was transported thither from other parts of the world by means of merchandise, seamen and animals. Its first appearance in Sweden cannot be determined with any certainty; but in France it began in the year 1414.” In contrast, 16th- and 17th-century descriptions of the disease and epidemics in Europe are documented frequently in the literature [46]. The absence of references to pertussis-like symptoms in the ancient literature has been taken as evidence that the association of *B. pertussis* with humans is of recent origin.

We propose that the association of *B. pertussis* with humans is, in fact, ancient but that the introduction of *B. pertussis* into Europe may be more recent. Complex IV strains showed a degree of diversity that was comparable to complex I strains (2.16 and 2.45, respectively), and thus, assuming that complex IV strains are primarily adapted to the human host, this association must be ancient. Parkhill et al. [10] previously estimated the time to the LCA of a *B. bronchiseptica* complex I strain (RB50) and *B. pertussis* to be 0.7 to 3.5 Mya, based on the mean number of synonymous substitutions per synonymous site of orthologous gene pairs. Our data indicate that current *B. pertussis* strains expanded clonally from the *B. pertussis*–*B. bronchiseptica* complex IV LCA 0.32 to 2.53 Mya, further supporting an ancient association of *B. pertussis* with humans. However, we cannot rule out the possibility that more recent human-associated ancestors of *B. pertussis* are extinct or undiscovered. Such recent ancestors would indicate a more recent origin of *B. pertussis.*

**Figure 5:**
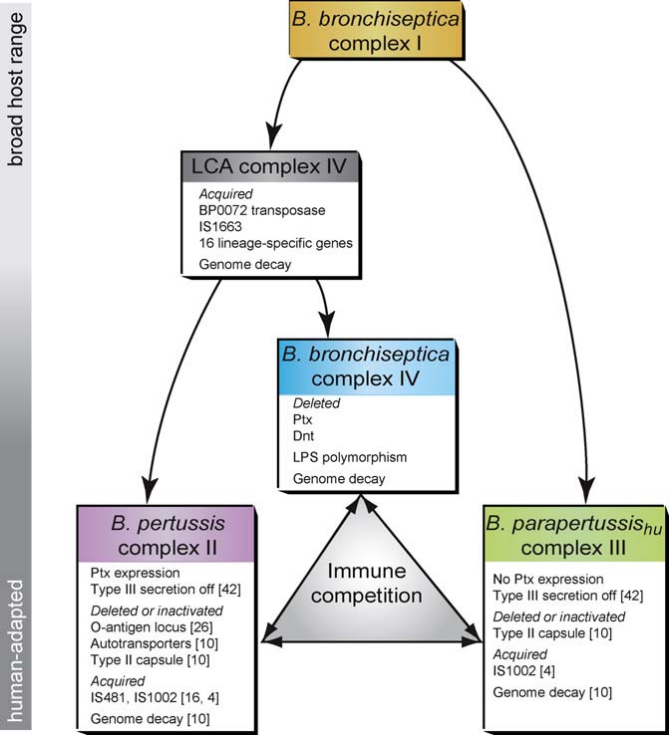
Model of the Evolution of the Mammalian Bordetellae The bar on the left indicates increasing degrees of adaptation to the human host. Arrows indicate descent; double arrows between complexes indicate possible within-host immune competition. In boxes, genetic events are shown that may have played a role in speciation and niche adaptation. Numbers between parentheses refer to references. See text for details.

Although it is tempting to speculate that the LCA of *B. pertussis* and *B. bronchiseptica* complex IV was associated with humans, the possibility remains that this association emerged after the split with *B. pertussis*. A possible evolutionary scenario (Figure 5) represents the adaptation of an ancestral *B. bronchiseptica* complex I strain to humans or their hominid ancestors. From this lineage, the LCA of *B. bronchiseptica* complex IV and *B. pertussis* evolved, subsequently giving rise to *B. bronchiseptica* complex IV and *B. pertussis*.

Recent emergence of a pathogenic clone from a more ancient human-associated progenitor species has been proposed as the mechanism for the origin of *Mycobacterium tuberculosis* [48]*.* Although previous genetic analysis had suggested that *M. tuberculosis* emerged as little as 20,000 years ago, phylogenetic analysis of *M. tuberculosis* and a closely related but more diverse group of smooth tubercle bacilli indicated that this more broadly defined species has been associated with hominids for up to 3 million years.

*Yersinia pestis,* the causative agent of plague, is a clone that evolved from *Y. pseudotuberculosis* 1,500 to 20,000 years ago, shortly before the first known pandemics of human plague, and its recent origin is further suggested by the complete lack of polymorphism in housekeeping genes [18]. Similarly, *B. pertussis* also shows limited diversity. However, in contrast to *Y. pestis,* which reveals absolutely no polymorphisms in housekeeping genes, we observed three STs in *B. pertussis*. This may suggest an older origin of *B. pertussis* compared to *Y. pestis,* although other factors, such as population size and bottlenecks, could also explain these differences. The most plausible explanation from our data is that the association of *B. pertussis* with humans originated in the LCA of *B. pertussis* and *B. bronchiseptica* complex IV. Based on that assumption, the apparent emergence of pertussis in Europe within the last 500 years may be attributable to import via travel or migration or to the recent acquisition by *B. pertussis* of the ability to cause more severe, whooping cough–like symptoms. Although most of the *B. bronchiseptica* complex IV strains in our collection were isolated from patients suspected to have pertussis, we know little of the severity of the symptoms caused by these strains. It is conceivable that *B. bronchiseptica* preceded *B. pertussis* in Europe and that its disease was not documented because of its relatively mild and nonspecific course.

The work presented here places the three sequenced mammalian *Bordetella* strains within a phylogenetic context, thereby facilitating rational selection of strains for further genomic sequencing. In particular, sequencing of one or more members of complex IV may shed more light on processes involved in host adaptation and immune competition. Further, the identification of a *B. bronchiseptica* lineage which circulates in human populations may be important for public health. In recent years, whole cell vaccines have been replaced by acellular vaccines comprised of one to five antigens derived from *B. pertussis* [49]. The acellular vaccines induce a less cross-reactive immune response compared to whole cell vaccines [50] and may therefore result in an increase in *B. parapertussis* and *B. bronchiseptica* infections in vaccinated human populations.

## Materials and Methods

### Bacterial strains.

A total of 132 *Bordetella* isolates were used in this study: 91 *B. bronchiseptica,* 9 *B. parapertussis_hu_,* 3 *B. parapertussis_ov_,* and 29 *B. pertussis* isolates (see Table S1). The three strains from which the genome sequence has been determined, *B. bronchiseptica* RB50*, B. pertussis* Tohama and *B. parapertussis* 12822 [10], were included. The collection included clinical isolates from humans and a broad range of animal species. Strains were grown on Bordet Gengou (BD, Franklin Lakes, New Jersey, United States) agar supplemented with 15% sheep blood at 37 °C for 2 to 5 days. Chromosomal DNA was isolated using the Wizard Genomic DNA Purification Kit (Promega, Madison, Wisconsin, United States), according to the manufacturers' protocol for Gram-negative bacteria.

### DNA sequencing.

The nucleotide sequences were determined for internal regions of seven housekeeping genes for all strains (http://pubmlst.org/bordetella [51]). The nucleotide sequence of the *prn* region encoding the extracellular domain of the surface-associated autotransporter pertactin, P.69, was determined for 116 strains, with the exclusion of the repeat regions 1 and 2 [35]. These regions are comprised of amino acids repeats and are highly polymorphic due to insertion or deletion of the repeat unit. Primer information is listed in Table S3.

### Detection of ISEs.

The distribution of IS*481,* IS*1001,* IS*1002,* and IS*1663* was determined for all strains using PCR amplification (see Table S1). For PCR amplification of IS*481,* IS*1001,* and IS*1002,* primers were used that have been described previously [4,52]. Primer characteristics are listed in Table S3.

### LPS SDS-PAGE and Western blotting.

BG-agar grown bacteria were harvested, boiled in 1× sample buffer (7.5% glycerol, 0.125 M Tris-HCl [pH 6.8], 1.5% SDS), and treated with proteinase K [33]. Tricine-SDS-PAGE was then performed in 4% stacking and 16% separating gels, as previously described by Lesse et al. [53]. Silver staining was performed as described by Tsai and Frasch [54]. LPS was transferred to PVDF membranes (Amersham Biosciences, Buckinghamshire, United Kingdom) and blocked with 0.5% (w/v) Protifar nonfat dried milk, 0.5% bovine serum albumin (w/v), and 0.1% Tween 20 in PBS. Immunoblotting was performed with monoclonal antibodies 36G3 and BL-8, directed against band A and band B LPS, respectively [31,32].

### Sequence data analysis.

Analysis of nucleotide sequence data was performed using Bionumerics software package version 4.0 beta 4 (Applied Maths, Sint-Martens-Latem, Belgium). The *Bordetella* MLST database can be accessed at http://pubmlst.org/bordetella [51].

For each locus in the MLST analysis, the allele sequences for all strains were trimmed to a uniform length, and an allele number was assigned to each unique allele sequence. The combination of the allele numbers at the seven loci defines the ST or allelic profile of each strain. Construction of trees based on allelic profiles may not accurately reflect the true genetic distance because both single and multiple nucleotide polymorphisms are given equal weight. Consequently, the degree of sequence difference between two alleles is not quantitatively reflected in the MLST profile. Conversely, tree construction based on concatenated allele sequences does not take into account the introduction of clustered multiple base substitutions due to a single recombinational event. As a result, trees based on MLST sequences often contain long branches, incorrectly suggesting a large genetic distance. Therefore, we used a method designated as split-MLST, in which each locus is split into a user-defined number of equally sized subloci (D. A. Diavatopoulos, P. Vauterin, L. Vauterin, F. R. Mooi, and L. M. Schouls, unpublished data). Using this method, the sensitivity of categorical clustering could be increased, without the perturbing effect of recombination. The topology of the tree appeared to vary if the number of subloci per MLST locus was lower than five. However, above the value four, increasing the number of subloci had no significant effect on the topology of the tree, and we therefore selected the lowest possible split value, five, resulting in a total of 35 subloci.

The genetic diversity for each complex was calculated using the Shannon-Weiner index of diversity (*H*) using the following formula:


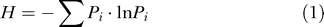


where *P_i_* is the frequency of the *i*th type [55].

For estimation of divergence times between complexes, we calculated the pairwise mean distance *(K_s_)* between alleles using DNASP 4.00 [56]. The divergence time was calculated using the following formula:


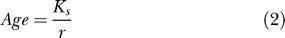


where *K_s_* is the number of synonymous substitutions per synonymous site and *r* is the molecular clock rate of *Escherichia coli* as determined by Whittam [19] or by Guttman and Dykhuizen [20]. We used these two rates to calculate a range of divergence times. The divergence time was first calculated for each combination of STs between complexes, and from these the averaged age between complexes was calculated.

### Comparative genomic hybridization.

The preparation of PCR product-based microarrays and the comparative genomic hybridization was performed essentially as described by Cummings et al. (12). This study employed a new array design that contained all of the probes from the first array plus 1,417 additional probes that brought the theoretical ORF coverage of these arrays up to 97.4% for *B. pertussis* Tohama, 98.5% for *B. bronchiseptica* RB50, and 97.9% for *B. parapertussis* 12822. Like the previously used array probes, these additional probes were PCR products with a size of less than 300 base pairs and amplified from the sequenced reference genomes with ORF-specific oligonucleotides (Illumina, San Diego, California, United States) designed with Microarray Architect (C. A. Cummings and D. A. Relman, unpublished data).

A total of 26 complex I and 13 complex IV strains were hybridized to the arrays. The genomic DNA of *B. bronchiseptica* was labeled with Cy5 and hybridized to the array in conjunction with a Cy3-labeled genomic DNA reference comprising the three sequenced mammalian *Bordetella* genomes (*B. pertussis* Tohama, *B. parapertussis* 12822, and *B. bronchiseptica* RB50). For the list of strains analyzed by CGH, see Table S1. Labeled probes were purified using the Cyscribe GFX Purification Kit (Amersham Biosciences, Freiburg, Germany) following the manufacturer's protocol for probes produced by the CyScribe First-Strand cDNA Labelling Kit. After purification, the test and reference-labeled DNA samples were concentrated to less than 8.5 μl using a Savant SpeedVac SVC-100H. The test and reference samples were combined and 150 μg of yeast tRNA (Invitrogen Life Technologies, San Diego, California, United States) was added to block nonspecific binding. The probe volume was adjusted to 24 μl with water and then 5.1 μl of 20× SSC (1× SSC = 0.15 M NaCl plus 0.015 M sodium citrate) and 0.9 μl of 10% sodium dodecyl sulfate (SDS) were added. Thirty microliters of the probe was added to the array and covered with a 25 × 40 mm No. 1 glass coverslip. Hybridization was performed in GeneMachines Hybchambers (Genomic Solutions, Ann Arbor, Michigan, United States) with 2× 30 μl of 3× SSC to maintain humidity and incubated at 65 °C overnight.

Arrays were washed in 0.5× SSC, 0.03% SDS for 30 s, 0.1× SSC, 0.01% SDS for 30 s, 0.05× SSC, 0.005% SDS for 1 min, and 0.025× SSC for 1 min. The first wash was performed at 65 °C, and the remaining washes were performed at room temperature. Slides were dried using a Quick-Dry Filtered Air Gun (Matrix Technologies Corporation, Hudson, New Hampshire, United States). Images were acquired on a PerkinElmer ScanArray 4000XL scanner using Scanarray Express software (PerkinElmer Life and Analytical Sciences, Inc., Boston, Massachusetts, United States). Images were analyzed with GenePix Pro software (Axon Instruments, Union City, California, United States).

Processed two-color array image data were submitted to an in-house microarray database. Data were extracted using filters to eliminate automatically and manually flagged spots and spots with very low background subtracted signal intensity (<150) in the reference channel. *B. bronchiseptica* complex I and complex IV enriched sequences were identified using the Significance Analysis for Microarrays software (SAM) [57]. The probes were analyzed using 26 complex I and 13 complex IV strains that were hybridized to the arrays. SAM analysis was run using the two-class option with KNN missing value imputation. In addition to a statistically significant difference, a 2-fold difference in mean signal intensity ratio for each probe was also required.

## Supporting Information

Dataset S1 CGH Data of the Mammalian Bordetellae (5.0 MB XLS)

Dataset S2 CGH Data of the Differentially Hybridizing Probes between Complexes I and IV as Identified by SAM (207 KB XLS)

Table S1 Characteristics of the Strains Used in the MLST Analysis (16 KB PDF)

Table S2 Probes That Hybridized Differentially to *B. bronchiseptica* Complex I and IV Genomes as Determined by SAM Analysis (19 KB PDF)

Table S3 Primer Characteristics for the Genes Used in Multilocus Sequence Typing, Pertactin Sequencing, and the Detection of the Insertion Sequence Elements (41 KB PDF)

### Accession Numbers

The nucleotide sequences of pertactin have been deposited in GenBank (http://www.ncbi.nlm.nih.gov/Genbank) under accession numbers DQ141700 through DQ141711 and DQ141713 through DQ141816.

## Abbreviations

CGH: comparative genomic hybridization
ISE: insertion sequence element
LCA: last common ancestor
LPS: lipopolysaccharide
mAb: monoclonal antibody
Mya: million years ago
MLST: multilocus sequence typing
MST: minimum spanning tree
ST: sequence type
