# Supplementary material for: Bordetella pertussis, the Causative Agent of Whooping Cough, Evolved from a Distinct, Human-Associated Lineage of B. bronchiseptica
Source: PLoS Pathog. 2005 Dec 30;1(4):e45. doi: 10.1371/journal.ppat.0010045 (PMC1323478; doi:10.1371/journal.ppat.0010045)
Supplement: Table S1 — (16 KB PDF) [file ppat.0010045.st001.pdf]

Table 1. Strain characteristics.

Strain Number	Species	Complex	CGH[a]	*Adk*	*FumC*	*GlyA*	*TyrB*	*Icd*	*PepA*	*Pgm*	ST	IS*481*	IS*1001*	IS*1002*	IS*1663*	Host	Country	Year	Original name	Reference[c]	Additional Information
B0015	BB[b]	I		2	4	4	1	1	4	2	7	-	+	-	-	pig	unknown	unknown	680	[1]	
B0084	BB	I	*	5	3	3	1	1	7	2	31	-	-	-	-	dog	unknown	unknown	PB383	W. Gaastra	
B0187	BB	I		2	2	4	1	3	4	2	14	-	-	-	-	unknown	South Africa	<1973	78	W. Gaastra	
B0188	BB	I		2	4	4	1	1	4	2	7	-	+	-	-	pig	South Africa	1991	608	W. Gaastra	
B0189	BB	I	*	5	3	3	1	1	3	2	27	-	-	-	-	dog	South Africa	1984	6635	M. Henton	
B0190	BB	I		2	4	4	1	1	4	2	7	-	-	-	-	pig	South Africa	1979	8136	M. Henton	
B0191	BB	I		2	4	4	1	1	4	2	7	-	+	-	-	rabbit	South Africa	1979	8171	M. Henton	
B0193	BB	I		2	4	4	1	1	4	2	7	-	+	-	-	rabbit	South Africa	<1973	Strachan	M. Henton	
B0194	BB	I		2	4	4	1	1	4	2	7	-	+	-	-	dog	USA	unknown	401 (or JM: MBORD 685)	J.M. Musser	
B0220	BB	I		2	4	4	1	1	4	2	7	-	+	-	-	pig	Australia	unknown	398	W. Gaastra	
B0222	BB	I		2	4	4	1	1	4	2	7	-	+	-	-	rabbit	Switzerland	unknown	417	W. Gaastra	
B0223	BB	I	*	2	2	4	1	3	4	2	14	-	-	-	-	guinea pig	Australia	1987	399	W. Gaastra	
B0224	BB	I	*	2	2	4	1	1	4	2	6	-	-	-	-	guinea pig	Germany	unknown	396	W. Gaastra	
B0225	BB	I		2	2	4	1	3	4	2	14	-	-	-	-	guinea pig	Ireland	unknown	428	W. Gaastra	
B0226	BB	I	*	2	3	4	1	3	3	2	5	-	-	-	-	cat	Netherlands	unknown	373	W. Gaastra	
B0227	BB	I	*	2	2	4	1	5	7	2	23	-	-	-	-	cat	Netherlands	unknown	385	W. Gaastra	
B0228	BB	I		2	4	4	1	1	4	2	7	-	+	-	-	cat	Netherlands	unknown	386	W. Gaastra	
B0229	BB	I		2	2	4	1	5	7	2	23	-	-	-	-	cat	Netherlands	unknown	387	W. Gaastra	
B0230	BB	I	*	2	2	4	1	1	4	2	6	+	-	-	-	horse	Denmark	unknown	406	[1]	
B0231	BB	I		2	4	4	1	1	4	2	7	-	+	-	-	mouse	USA	unknown	390	W. Gaastra	
B0233	BB	I		2	4	4	1	1	4	2	7	-	+	-	-	cow	Ireland	unknown	415	W. Gaastra	
B0234	BB	I		2	4	4	1	1	4	2	7	-	+	-	-	rabbit	Denmark	unknown	389	W. Gaastra	
B0236	BB	I		2	3	4	1	1	4	2	10	-	-	-	-	rabbit	USA	unknown	395	W. Gaastra	
B0237	BB	I	*	2	6	4	1	1	4	2	12	-	-	-	-	rabbit	Denmark	unknown	405	W. Gaastra	
B0238	BB	I	*	5	3	3	1	1	3	2	27	-	-	-	-	dog	Netherlands	unknown	207	W. Gaastra	
B0242	BB	I	*	2	4	4	1	1	4	2	7	-	+	-	-	pig	USA	unknown	688	[1]	
B0246	BB	I		2	3	3	1	1	3	2	4	-	-	-	-	pig	Netherlands	unknown	548	[1]	
B0247	BB	I		2	4	4	1	1	4	2	7	-	+	-	-	pig	Australia	unknown	677	[1]	
B0248	BB	I		2	4	4	1	1	4	2	7	-	-	-	-	guinea pig	USA	unknown	669	[1]	
B0251	BB	I	*	2	2	4	1	5	7	2	23	-	-	-	-	dog	Netherlands	unknown	355	J.M. Musser	
B0254	BB	I	*	2	4	4	1	1	4	2	7	-	-	-	-	rabbit	Netherlands	1987	372	J.M. Musser	
B0258	BB	I	*	2	3	4	1	1	4	2	10	-	-	-	-	rabbit	USA	unknown	705	[1]	
B0260	BB	I	*	2	2	4	1	5	7	2	23	-	-	-	-	cat	Denmark	unknown	723	[1]	
B0261	BB	I		2	6	4	1	1	4	2	12	-	-	-	-	rabbit	Denmark	unknown	730	[1]	
B0263	BB	I		2	4	4	1	1	4	2	7	-	+	-	-	pig	Australia	unknown	676	[1]	
B0505	BB	I	*	2	6	4	1	1	4	2	12	-	-	-	-	human	Netherlands	1997	9700219	This study	
B1813	BB	I		2	6	4	1	1	4	2	12	-	-	-	-	unknown	unknown	unknown		This study	
B1965	BB	I	*	2	3	4	1	1	4	2	10	-	-	-	-	dog	unknown	unknown	590	[1]	
B1966	BB	I	*	5	3	3	1	1	3	2	27	-	-	-	-	dog	unknown	unknown	599	[1]	
B1967	BB	I	*	2	3	3	1	1	3	2	4	-	-	-	-	dog	unknown	unknown	601	[1]	
B1970	BB	I		2	4	4	1	1	4	2	7	-	+	-	-	pig	unknown	unknown	545	[1]	
B1971	BB	I		2	3	4	1	1	4	2	10	-	-	-	-	rabbit	unknown	unknown	671	[1]	
B1972	BB	I	*	2	3	4	1	3	3	2	5	-	-	-	-	cat	unknown	unknown	782	[1]	
B1973	BB	I		2	2	7	1	1	7	2	32	-	-	-	-	monkey	unknown	unknown	902	[1]	
B1975	BB	I	*	2	2	4	1	1	4	2	6	+	-	-	-	horse	unknown	unknown	982	[1]	
B1976	BB	I		2	6	4	1	1	4	2	12	-	-	-	-	rabbit	USA		RB50	[2, 3]	Genome sequenced
B1977	BB	I		2	6	4	1	1	4	2	12	-	-	-	-	guinea pig	USA	unknown	GP1SN	[4]	
B1978	BB	I		2	6	4	1	1	4	2	12	-	-	-	-	human	USA	unknown	JC100	J. Miller	
B1985	BB	I	*	2	2	4	1	1	4	2	6	-	-	-	-	seal	North Sea	1988	M861/99/1	G. Foster	
B1986	BB	I	*	2	2	4	1	1	4	2	6	-	-	-	-	seal	North Sea	1997	M2936/97/3	G. Foster	
B1987	BB	I	*	2	2	4	1	1	7	2	33	-	-	-	-	seal	Caspian Sea	2000	M85/00/1	G. Foster	
B2103	BB	I		5	3	3	1	1	3	2	27	-	-	-	-	human	Netherlands	1997	97-219	This study	
B2104	BB	I	*	2	2	4	1	5	7	2	23	-	-	-	-	human	Netherlands	1995	95-638	This study	
B2105	BB	I		5	3	3	1	1	3	2	27	-	-	-	-	human	Netherlands	1995	95-646	This study	
B2108	BB	I		2	4	4	1	1	4	2	7	-	+	-	-	human	Netherlands	1996	96-90	This study	
B2109	BB	I		2	6	4	1	1	4	2	12	-	-	-	-	human	Netherlands	1997	97-219	This study	
B2110	BB	I		2	2	4	1	5	7	2	23	-	-	-	-	human	Netherlands	1997	97-772	This study	
B2112	BB	I	*	2	4	4	1	1	4	2	7	-	+	-	-	human	Netherlands	1998	98-061	This study	
B2113	BB	I		5	3	3	1	1	3	2	27	-	-	-	-	human	Netherlands	1998	98-534	This study	
B2115	BB	I		2	2	1	1	6	7	2	26	-	-	-	-	human	Netherlands	2000	00-098	This study	
B2116	BB	I	*	2	3	3	1	1	3	2	4	-	-	-	-	human	Netherlands	2000	00-257	This study	
B2489	BB	I		2	4	4	1	1	4	2	7	-	+	-	-	guinea pig	USA	unknown	SBL-F4016	G. Sanden	
B2493	BB	I		5	3	3	1	1	3	2	27	-	-	-	-	human	USA	unknown	MO108	G. Sanden	
B2497	BB	I		2	4	4	1	1	4	2	7	-	+	-	-	human	USA	unknown	MO289	G. Sanden	
B2498	BB	I		2	2	4	1	5	7	2	23	-	-	-	-	human	USA	unknown	MO1256	G. Sanden	
B2499	BB	I		2	4	4	1	1	4	2	7	-	+	-	-	unknown	USA	unknown	MOQC	G. Sanden	
B2500	BB	I		5	3	3	1	1	3	2	27	-	-	-	-	human	USA	1998	99-R-0311	G. Sanden	
B2501	BB	I		5	3	3	1	1	3	2	27	-	-	-	-	human	USA	1998	99-R-0313	G. Sanden	
B2503	BB	I		2	4	4	1	1	4	2	7	-	-	-	-	human	USA	1999	99-R-0448	G. Sanden	
B2504	BB	I		2	6	4	1	1	4	2	12	-	-	-	-	human	USA	2000	00-P-1465	G. Sanden	
B2505	BB	I		2	6	4	1	1	4	2	12	-	-	-	-	human	USA	2000	00-P-1466	G. Sanden	
B2507	BB	I		5	3	3	1	1	3	2	27	-	-	-	-	human	USA	2001	00-P-2796	G. Sanden	
B2508	BB	I		5	3	3	1	1	3	2	27	-	-	-	-	human	USA	2001	01-P-0075	G. Sanden	
B2509	BB	I		2	6	4	1	1	4	2	12	-	-	-	-	human	USA	2001	01-R-0468	G. Sanden	
B2510	BB	I		5	3	3	1	1	3	2	27	-	-	-	-	human	USA	2001	01-P-0557	G. Sanden	
B2511	BB	I		5	3	3	1	1	3	2	27	-	-	-	-	human	USA	2001	01-R-0572	G. Sanden	
B0203	BPP-OV	I		2	2	5	1	1	5	3	16	-	+	-	-	sheep	New Zealand	unknown	927	B.W. Manktelow	
B0204	BPP-OV	I		2	2	5	1	1	5	3	16	-	+	-	-	sheep	New Zealand	unknown	928	B.W. Manktelow	
B0207	BPP-OV	I		2	2	5	1	1	5	3	16	-	+	-	-	sheep	New Zealand	unknown	585	B.W. Manktelow	
B0006	BP	II	*	1	1	1	1	1	1	1	1	+	-	+	+	human	Netherlands	1950	509		Dutch vaccine strain
B0213	BP	II	*	1	1	1	1	1	1	1	1	+	-	+	+	human	Japan	1952	Tohama I	[3, 6]	Genome sequenced
B0336	BP	II	*	1	1	1	3	1	1	1	2	+	-	+	+	human	Netherlands	1952	Hp291	This study	
B0352	BP	II	*	1	1	1	3	1	1	1	2	+	-	+	+	human	Netherlands	1994	9400526	This study	
B0402	BP	II		1	1	1	1	1	1	1	1	+	-	+	+	human	China	1957	940225	This study	
B0403	BP	II		1	1	1	3	1	1	1	2	+	-	+	+	human	China	1957		This study	
B0441	BP	II	*	1	1	1	1	1	1	1	1	+	-	+	+	human	Netherlands	1952	459	This study	
B0540	BP	II	*	1	1	1	3	1	1	1	2	+	-	+	+	human	Netherlands	1994	9400450	This study	
B0558	BP	II	*	1	1	1	3	1	1	1	2	+	-	+	+	human	Netherlands	1949	Hp53	This study	
B0564	BP	II	*	1	1	1	1	1	1	1	1	+	-	+	+	human	Netherlands	1950	Hp199	This study	
B0604	BP	II	*	1	1	1	3	1	1	1	2	+	-	+	+	human	Netherlands	1995	9500451	This study	
B0620	BP	II		1	1	1	3	1	1	1	2	+	-	+	+	human	USA	1956	134 lederle	[8]	
B0782	BP	II	*	1	1	1	3	1	1	1	2	+	-	+	+	human	Netherlands	1996	9600427	This study	
B0939	BP	II	*	1	1	1	3	1	1	1	2	+	-	+	+	human	Netherlands	1996	9600498	This study	
B1054	BP	II		1	1	1	3	1	1	1	2	+	-	+	+	human	Japan	1996	603	[1]	
B1055	BP	II		1	1	1	3	1	1	1	2	+	-	+	+	human	Japan	1996	604	[1]	
B1063	BP	II		1	1	1	3	1	1	1	2	+	-	+	+	human	Finland	1996	211/PRCB (44)	J. Mertsola	
B1121	BP	II	*	2	1	1	1	1	1	1	24	+	-	+	+	human	USA	1947	18-323	N. Guiso	Mouse challenge strain
B1317	BP	II		1	1	1	3	1	1	1	2	+	-	+	+	human	Netherlands	1965	K64	This study	
B1325	BP	II		1	1	1	3	1	1	1	2	+	-	+	+	human	Australia	1997	7A-14	P. McIntyre	
B1412	BP	II		1	1	1	3	1	1	1	2	+	-	+	+	human	Senegal	1992	A156	G. Sanden	
B1419	BP	II		1	1	1	3	1	1	1	2	+	-	+	+	human	USA	1997	A713	G. Sanden	
B1422	BP	II		1	1	1	3	1	1	1	2	+	-	+	+	human	USA	1996	A916	G. Sanden	
B1424	BP	II		1	1	1	3	1	1	1	2	+	-	+	+	human	Mexico	1997	A951	G. Sanden	
B1443	BP	II		1	1	1	3	1	1	1	2	+	-	+	+	human	Mexico	1996	A944	G. Sanden	
B1617	BP	II		1	1	1	3	1	1	1	2	+	-	+	+	human	Australia	1998	980 2837	P. McIntyre	
B1979	BP	II		1	1	1	3	1	1	1	2	+	-	+	+	human	unknown	unknown	CS	[4]	
B1980	BP	II		1	1	1	1	1	1	1	1	+	-	+	+	human	unknown	unknown	VM	[4]	

B1981	BP	II		1	1	1	3	1	1	1	2	+	-	+	+	human	unknown	unknown	GMT1	[4]	
B0024	BPP-HU	III		2	2	2	2	2	2	5	19	-	+	+	-	human	Netherlands	1985	1206	[1]	
B0266	BPP-HU	III		2	2	2	2	2	2	5	19	-	+	+	-	human	Finland	1982	FL2	[1, 5]	
B0267	BPP-HU	III		2	2	2	2	2	2	5	19	-	+	+	-	human	Finland	1982	FL3	[5]	
B0273	BPP-HU	III		2	2	2	2	2	2	5	19	-	+	+	-	human	USA	unknown	531	[1]	
B0285	BPP-HU	III		2	2	2	2	2	2	5	19	-	+	+	-	human	Japan	1985	167	[7]	
B0291	BPP-HU	III		2	2	2	2	2	2	5	19	-	+	+	-	human	Japan	1985	166	[7]	
B0319	BPP-HU	III		2	2	2	2	2	2	5	19	-	+	+	-	human	USA	1981	81A-3084	This study	
B1318	BPP-HU	III		1	2	2	2	2	2	5	30	-	+	+	-	human	Netherlands	1965	K65/K75	This study	
B2002	BPP-HU	III		2	2	2	2	2	2	5	19	-	+	+	-	human	Germany	unknown	12822	[3]	Genome sequenced
B0232	BB	IV	*	3	2	6	2	4	6	4	18	-	-	-	-	human	Germany	unknown	397	W. Gaastra	
B0243	BB	IV	*	4	2	1	2	2	8	7	28	-	-	-	+	pig	Ukraine	unknown	654	[1]	
B0259	BB	IV	*	4	2	6	4	1	8	8	29	-	-	-	+	turkey	USA	unknown	707	[1]	
B1968	BB	IV	*	3	5	6	2	1	6	6	22	-	-	-	+	dog	unknown	unknown	591	[1]	
B1969	BB	IV	*	3	2	6	2	4	6	4	18	-	-	-	-	human	unknown	unknown	675	[1]	
B2114	BB	IV	*	3	2	1	2	4	6	6	25	-	-	-	+	human	Netherlands	1999	99-003	This study	
B2490	BB	IV	*	3	5	1	5	7	8	6	9	-	-	-	+	human	USA	unknown	SBL-F6116	G. Sanden	
B2491	BB	IV	*	3	2	8	2	4	8	9	8	-	-	-	+	human	USA	unknown	SBL-F6368	G. Sanden	
B2492	BB	IV	*	6	2	9	4	1	9	7	21	-	-	-	-	human	USA	1996	GA96-01	G. Sanden	
B2494	BB	IV	*	4	2	1	2	8	8	6	15	-	-	-	+	human	USA	unknown	MO149	G. Sanden	
B2495	BB	IV	*	4	5	1	2	4	8	6	17	-	-	-	+	human	USA	unknown	MO211	G. Sanden	
B2496	BB	IV	*	3	2	1	2	4	6	7	3	-	-	-	+	human	USA	unknown	MO275	G. Sanden	
B2506	BB	IV	*	3	5	1	5	7	6	6	34	-	-	-	+	human	USA	2000	00-P-2730	G. Sanden	
B2586	BB	IV		3	2	1	2	4	6	7	3	-	-	-	+	human	Argentina	>1998	203	D. Hozbor	
B2588	BB	IV		3	5	10	2	4	8	6	35	-	-	-	+	human	Argentina	>1998	5256	D. Hozbor	

[a] Strains used in the comparative genomic comparison

[b] Abbreviations: BB=*B. bronchiseptica*; BPP-OV= *B. parapertussis*-ovine; BPP-HU=*B. parapertussis*-human; BP=*B. pertussis*

[c] Numbers between brackets indicate references:

[1] van der Zee A, Mooi F, van Embden J, Musser J (1997) Molecular evolution and host adaptation of Bordetella spp.: phylogenetic analysis using multilocus enzyme electrophoresis and typing with three insertion sequences. J Bacteriol 179: 6609-6617.

[2] Cotter PA, Miller JF (1994) BvgAS-mediated signal transduction: analysis of phase-locked regulatory mutants of Bordetella bronchiseptica in a rabbit model. Infect Immun 62: 3381-3390.

[3] Parkhill J, Sebaihia M, Preston A, Murphy LD, Thomson N, Harris DE, Holden MT, Churcher CM, Bentley SD, Mungall KL, Cerdeno-Tarraga AM, Temple L, James K, Harris B, Quail MA, Achtman M, Atkin R, Baker S, Basham D, Bason N, Cherevach I, Chillingworth T, Collins M, Cronin A, Davis P, Doggett J, Feltwell T, Goble A, Hamlin N, Hauser H, Holroyd S, Jagels K, Leather S, Moule S, Norberczak H, O'Neil S, Ormond D, Price C, Rabbinowitsch E, Rutter S, Sanders M, Saunders D, Seeger K, Sharp S, Simmonds M, Skelton J, Squares R, Squares S, Stevens K, Unwin L, Whitehead S, Barrell BG, Maskell DJ (2003) Comparative analysis of the genome sequences of Bordetella pertussis, Bordetella parapertussis and Bordetella bronchiseptica. Nat Genet

[4] Martinez dT, Miller JF, Cotter PA (1996) Comparative analysis of the virulence control systems of Bordetella pertussis and Bordetella bronchiseptica. Mol Microbiol 22: 895-908.

[5] Mertsola J (1985) Mixed outbreak of Bordetella pertussis and Bordetella parapertussis infection in Finland. Eur J Clin Microbiol 4: 123-128.

[6] Sato Y, Arai H (1972) Leucocytosis-promoting factor of Bordetella pertussis. I. Purification and characterization. Infect Immun 6: 899-904.

[7] Iwata S, Aoyama T, Goto A, Iwai H, Sato Y, Akita H, Murase Y, Oikawa T, Iwata T, Kusano S, . (1991) Mixed outbreak of Bordetella pertussis and Bordetella parapertussis in an apartment house. Dev Biol Stand 73: 333-341.

[8] Cherry JD, Mortimer EA, Hackell JG, Scott JV (1991) Clinical trials in the United States and Japan with the Lederle-Takeda and Takeda acellular pertussis-diphtheria-tetanus (APDT) vaccines. The Multicenter APDT Vaccine Study Groups. Dev Biol Stand 73: 51-58.
