# Supplementary material for: Bordetella pertussis, the Causative Agent of Whooping Cough, Evolved from a Distinct, Human-Associated Lineage of B. bronchiseptica
Source: PLoS Pathog. 2005 Dec 30;1(4):e45. doi: 10.1371/journal.ppat.0010045 (PMC1323478; doi:10.1371/journal.ppat.0010045)
Supplement: Table S2 — (19 KB PDF) [file ppat.0010045.st002.pdf]

Table S2. Probes that hybridized differentially to *B. bronchiseptica* complex I and IV genomes as determined by SAM analysis

Probe ID	Probe species[a]	BB ORF[b]	BP ORF[b]	BPP ORF[b]	Mean fold change[c]	Gene name and product[d]	Function[e]	Multifun
Overrepresented in complex IV								
5046	BB	0382			2.49	hypothetical protein		Unknown function, no known homologs
2393	BP	0408	0227	0406	2.09	*psiF;* phosphate starvation-inducible protein precursor		Adaptation
1373	BP	0473	0425	0473	2.75	probable LysR-family transcriptional regulator		Global regulatory functions
3742	BP	1366	1054	1150	5.12	*prn;* pertactin precursor	adhesin	Pathogenicity islands/determinants
2624	BP	1649	2224	2251	2.13	*bapA;* putative autotransporter		Unknown function, no known homologs
303	BP	1847	2017	2397	3.00	putative haloacid-type dehydrogenase		Not classified (including putative assignments)
3756	BP	2031	2741	2588	4.48	putative TetR-family transcriptional regulator		Global regulatory functions
2690	BP	[3158][f]	[1652]	[2837]	3.12	intergenic region		
3530	BP	3291	1201		3.00	*tcfA;* tracheal colonization factor precursor	adhesin	Pathogenicity islands/determinants
1925	BP	4137	0072		9.43	transposase		Transposon-related functions
3734	BP	4708	0632		2.15	putative pimeloyl-CoA synthetase		
2343	BP		0207		13.59	putative aldehyde dehydrogenase		Not classified (including putative assignments)
1655	BP		0328		2.89	conserved hypothetical protein		Conserved in other organisms
1654	BP		0329		11.24	putative coenzyme A ligase		Not classified (including putative assignments)
3578	BP		[0332]		4.15	intergenic region		
1618	BP		0703		11.82	TonB-dependent receptor protein (pseudogene)	iron uptake	Fe storage
1664	BP		[2182]		2.09	intergenic region		
1665	BP		2183		3.13	conserved hypothetical protein		Conserved in other organisms
2563	BP		2306		7.46	putative sodium:alanine symporter		Transport/binding proteins
2747	BP		2603		2.05	hypothetical protein		Unknown function, no known homologs
3656	BP		[2618]		5.62	intergenic region		
1231	BP		2622		4.09	HipA-like protein		Conserved in other organisms
1229	BP		2623		3.84	DNA-binding protein		Not classified (including putative assignments)
3443	BP		2853		2.23	probable short chain dehydrogenase		Pool, multipurpose conversions of intermed. met'm
1954	BP		2854		3.40	probable short chain dehydrogenase		Pool, multipurpose conversions of intermed. met'm
1955	BP		2855		3.77	membrane transport protein		Transport/binding proteins
3535	BP		2857		2.88	putative LysR-family transcriptional regulator		Global regulatory functions
3671	BP		multiple		13.68	IS1663 transposon	transposase	
3670	BP		multiple		12.24	IS1663 transposon	transposase	
6087	BPP			2077	12.08	conserved hypothetical protein		Conserved in other organisms
6088	BPP			2078	12.87	conserved hypothetical protein		Conserved in other organisms
Underrepresented in complex IV								
5535	BB	0022		0022	0.33	putative metal transporter		
5536	BB	0022		0022	0.41	putative metal transporter		
5184	BB	[0109]			0.18	intergenic region		
7004	BB	0110			0.18	putative adhesin	adhesin	Pathogenicity islands/determinants
5178	BB	0124			0.35	putative UDP-glucose 4-epimerase		Not classified (including putative assignments)
7006	BB	0128			0.17	hypothetical protein		Unknown function, no known homologs
5173	BB	0130		0130	0.38	*wbmO;* hypothetical protein		
5172	BB	0131		0131	0.46	*wbmN;* ABC transporter ATP-binding protein		
5163	BB	0138		0138	0.17	*wbmG;* nucleotide sugar epimerase/dehydratase	LPS O-antigen biosynthesis	
5160	BB	0140		0140	0.33	*wbmE;* putative exported protein		
5159	BB	0141		0141	0.44	*wbmD;* putative membrane protein		
5157	BB	0142		0142	0.48	*wbmC;* putative glutamine amidotransferase		
5154	BB	0144		0144	0.46	*wbmA;* putative glycosyl transferase		
5209	BB	0214		0210	0.36	AraC family transcriptional regulator		
5207	BB	0216		0212	0.10	conserved hypothetical protein		
5051	BB	0376		0373	0.46	putative membrane protein		
5050	BB	0377		0374	0.49	putative membrane protein		
5022	BB	[0449]		[0448]	0.07	intergenic region		
3085	BP	0466	0433	0466	0.50	probable LysR-family transcriptional regulator		Global regulatory functions
1366	BP	0467	0432	0467	0.44	conserved hypothetical protein		Conserved in other organisms
7033	BB	0471	0427	0471	0.45	putative exported protein		Periplasmic/exported/lipoproteins
7038	BB	0477		0477	0.18	conserved hypothetical protein		
7039	BB	0478		0478	0.11	conserved hypothetical protein		
7040	BB	0479		0479	0.13	hypothetical protein		
6030	BB	[0481]		0482	0.30	hypothetical protein (BPP) or intergenic region (BB)		Unknown function, no known homologs
6037	BB	0486		0487	0.47	hypothetical protein		Unknown function, no known homologs
6038	BB	0488		0488	0.34	*virB6;* plasmid-related protein		Plasmid-related functions
7048	BB	0490			0.16	putative membrane protein	plasmid- or phage-like region	Inner membrane
7049	BB	0491			0.11	*virB5;* plasmid-related exported protein		Plasmid-related functions
7050	BB	0492			0.02	hypothetical protein		Unknown function, no known homologs
7051	BB	0493			0.18	*virD2;* plasmid-related protein		Plasmid-related functions
7052	BB	0494			0.03	hypothetical 20.3 kDa protein, plasmid-related protein		Plasmid-related functions
7054	BB	0496		0488	0.10	plasmid-related protein ; (BB) hypothetical protein		Unknown function, no known homologs
7055	BB	0497			0.05	hypothetical protein		Unknown function, no known homologs
7056	BB	0498			0.28	hypothetical protein		Unknown function, no known homologs
7057	BB	0499		0494	0.12	hypothetical protein		
7058	BB	0500			0.29	putative integrase		Phage-related functions and prophages
7064	BB	0527			0.02	conserved hypothetical protein		Conserved in other organisms
7065	BB	0528			0.11	putative DNA-binding protein		Not classified (including putative assignments)
7066	BB	0529			0.12	hypothetical protein		Unknown function, no known homologs
7071	BB	0534		0529	0.46	putative exported protein		
7108	BB	0571		0565	0.48	putative branched-chain amino acid transport ATP-binding protein		
7124	BB	0587		0581	0.12	conserved hypothetical protein		
7180	BB	0652			0.16	putative exported protein		Periplasmic/exported/lipoproteins
7181	BB	0653			0.11	putative 3-hydroxyisobutyrate dehydrogenase		Not classified (including putative assignments)
7182	BB	0654			0.09	LysR-family transcriptional regulator		Global regulatory functions
7245	BB	0718			0.36	*bcpA;* carboxyvinyl-carboxyphosphonate phosphorylmutase		Not classified (including putative assignments)
7247	BB	0720			0.49	putative membrane protein		Inner membrane
7324	BB	0815		0729	0.42	putative outer membrane protein		
7330	BB	0821		0735	0.25	autotransporter		
5218	BB	0908			0.42	putative helicase		DNA - replication, repair, restr./modific'n
5219	BB	0909			0.09	conserved hypothetical protein		Conserved in other organisms
5223	BB	0914	3112		0.23	putative integrase (BP pseudogene)		Conserved in other organisms
5224	BB	0915			0.13	conserved hypothetical protein		Conserved in other organisms
2267	BP	0924	3454	0830	0.48	putative lipoprotein		Periplasmic/exported/lipoproteins
2266	BP	0925	3455	0831	0.46	putative taurine dioxygenase		Amines
7351	BB	0981		0888	0.46	probable acyl-CoA dehydrogenase		
7352	BB	0982		0889	0.08	conserved hypothetical protein		
7353	BB	0983		0890	0.10	putative exported protein		
7354	BB	0984		0891	0.22	probable LysR-family transcriptional regulator		
7391	BB	1021			0.14	putative membrane protein		Inner membrane
7393	BB	1024			0.42	LysR-family transcriptional regulator		Global regulatory functions
7395	BB	1026			0.46	putative exported protein		Periplasmic/exported/lipoproteins
7397	BB	1028			0.38	putative exported protein		Periplasmic/exported/lipoproteins
7422	BB	1054			0.50	LysR-family transcriptional regulator		Global regulatory functions
7467	BB	1100			0.05	*mgtA;* Mg(2+) transport ATPase		Cations
7468	BB	1101			0.49	two component sensor kinase		Global regulatory functions
7567	BB	1221		1008	0.40	hypothetical protein		
5431	BB	1342		1126	0.47	*acnA;* aconitate hydratase		
5429	BB	1344		1128	0.50	probable hydrolase		
8166	BB	1366	1054	1150	0.44	*prn;* pertactin precursor	adhesin	Pathogenicity islands/determinants
6060	BPP	1366	1054	1150	0.49	*prn;* pertactin precursor	adhesin	Pathogenicity islands/determinants
7633	BB	1470			0.07	conserved hypothetical protein		Conserved in other organisms
7634	BB	1471			0.01	putative exported protein		Periplasmic/exported/lipoproteins
7635	BB	1472			0.01	conserved hypothetical protein		Conserved in other organisms
7636	BB	1473			0.01	putative oxidoreductase		Not classified (including putative assignments)
7637	BB	1474			0.01	putative DNA-binding protein		Not classified (including putative assignments)
7657	BB	1494		2098	0.44	conserved hypothetical protein		
7658	BB	1495		2099	0.43	Putative transcriptional regulator		
7659	BB	1496		2100	0.43	Putative short chain dehydrogenase		
7660	BB	1497		2101	0.19	putative exported protein		
7661	BB	1498		2102	0.06	Putative hydrolase		
7662	BB	1499		2103	0.14	Putative monoxygenase		
7663	BB	1500		2104	0.04	Putative 3-hydroxybutyryl-CoA dehydrogenase		
5444	BB	1606		2209	0.46	Putative oxidoreductase ; (Bpp) Pseudogene		
5443	BB	1648			0.27	putative acetyltransferase		Not classified (including putative assignments)
5576	BB	1665			0.08	phage-related putative exported protein		Phage-related functions and prophages
5531	BB	1666			0.32	phage-related hypothetical protein		Phage-related functions and prophages
5527	BB	1670			0.19	phage-related hypothetical protein		Phage-related functions and prophages
5526	BB	1671			0.07	phage-related hypothetical protein		Phage-related functions and prophages
5524	BB	1673			0.23	phage-related hypothetical protein		Phage-related functions and prophages
5518	BB	1681			0.20	phage-related hypothetical protein		Phage-related functions and prophages
5515	BB	1684			0.26	phage-related hypothetical protein	prophage	Phage-related functions and prophages
7731	BB	1691			0.09	phage-related hypothetical protein		Phage-related functions and prophages
5502	BB	1698			0.23	phage-related conserved hypothetical protein		Phage-related functions and prophages
5500	BB	1701			0.04	phage-related conserved hypothetical protein		Phage-related functions and prophages
5497	BB	1706			0.05	putative tail component of prophage		Phage-related functions and prophages
5496	BB	1708			0.20	phage-related hypothetical protein		Phage-related functions and prophages
5027	BB	1797		2346	0.48	Putative TetR-family regulatory protein ; (Bpp) Pseudogene		
5566	BB	[1903]		[2455]	0.45	intergenic region		
5025	BB	1904		2456	0.18	Putative sigma-70 factor, ECF subfamily		
5026	BB	1905		2457	0.17	putative extracellular heme-binding protein		
7758	BB	2031	2741	2588	0.43	putative TetR-family transcriptional regulator		Global regulatory functions
7777	BB	2188			0.04	phage-related conserved hypothetical protein		Phage-related functions and prophages
5019	BB	2189			0.29	phage-related conserved hypothetical protein		Phage-related functions and prophages
7778	BB	2190			0.02	phage-related hypothetical protein		Phage-related functions and prophages

12	BP	2191	0508		0.09	phage-related exonuclease		Phage-related functions and prophages
10	BP	2194	0511		0.27	phage-related hypothetical protein		Transport/binding proteins
9	BP	2196	0513		0.26	phage-related hypothetical protein		Ribosomal proteins - synthesis, modification
2189	BP	2201	3388		0.09	phage-related putative membrane protein		Unknown function, no known homologs
7780	BB	2201	3387		0.15	Pseudogene; (BB, BB) phage-related hypothetical protein		Phage-related functions and prophages
7781	BB	2203			0.10	phage-related conserved hypothetical protein		Phage-related functions and prophages
7782	BB	2204			0.11	phage-related conserved hypothetical protein		Phage-related functions and prophages
7783	BB	2205			0.08	phage-related conserved hypothetical protein		Phage-related functions and prophages
7785	BB	2211			0.32	phage-related hypothetical protein		Phage-related functions and prophages
5090	BB	2212			0.30	phage-related hypothetical protein		Phage-related functions and prophages
5089	BB	2213	3385		0.20	phage-related putative DNA binding protein		Conserved in other organisms
1356	BP	2214	3384		0.18	putative phage terminase		Unknown function, no known homologs
5088	BB	2214	3384		0.22	putative phage terminase		Unknown function, no known homologs
5087	BB	2215	3383		0.33	phage-related conserved hypothetical protein		Plasmid-related functions
1357	BP	2215	3383		0.21	phage-related conserved hypothetical protein		Plasmid-related functions
1358	BP	2216	3382		0.28	phage-related conserved hypothetical protein		Unknown function, no known homologs
5086	BB	2216	3382		0.25	phage-related conserved hypothetical protein		Unknown function, no known homologs
1359	BP	2217	3381		0.30	phage-related hypothetical protein	prophage	Unknown function, no known homologs
5085	BB	2218	3380		0.21	phage-related hypothetical protein		Unknown function, no known homologs
1360	BP	2219	3379		0.12	phage-related conserved hypothetical protein		Unknown function, no known homologs
8066	BB	2220	3376		0.33	phage-related hypothetical protein		Unknown function, no known homologs
7787	BB	2220	3376		0.32	phage-related hypothetical protein		Unknown function, no known homologs
2137	BP	2224	3372		0.35	phage-related conserved hypothetical protein		Unknown function, no known homologs
8064	BB	2224	3372		0.45	phage-related conserved hypothetical protein		Unknown function, no known homologs
2138	BP	2225	3371		0.33	phage-related conserved hypothetical protein		Conserved in other organisms
324	BP	2226	3370		0.33	phage-related conserved hypothetical protein		Conserved in other organisms
325	BP	2227	3369		0.20	phage-related conserved hypothetical protein		Unknown function, no known homologs
326	BP	2228	3368		0.30	phage-related conserved hypothetical protein		Unknown function, no known homologs
327	BP	2229	3367		0.16	phage-related conserved hypothetical protein		Unknown function, no known homologs
328	BP	2230	3366		0.04	phage hk97 tail length tape measure-related protein		Phage-related functions and prophages
329	BP	2231	3365		0.15	phage-related conserved hypothetical protein		Phage-related functions and prophages
333	BP	2234	3362		0.18	phage-related conserved hypothetical protein		Phage-related functions and prophages
334	BP	2235	3361		0.35	phage-related hypothetical protein		Unknown function, no known homologs
7795	BB	2237	3359		0.14	phage-related hypothetical protein		Phage-related functions and prophages
335	BP	2238	3358		0.21	phage-related hypothetical protein		Phage-related functions and prophages
336	BP	2239	3357		0.12	phage-related conserved hypothetical protein		Phage-related functions and prophages
337	BP	2240	3356		0.06	putative phage lysozyme		Phage-related functions and prophages
339	BP	2242	3354		0.32	phage-related hypothetical protein		Unknown function, no known homologs
50	BP	2303	1112	1234	0.37	*bipA;* putative outer membrane ligand binding protein		Pathogenicity islands/determinants
7802	BB	2303	1112	1234	0.15	*bipA;* putative outer membrane ligand binding protein		Pathogenicity islands/determinants
6065	BPP	2312	2667	1243	0.38	*fhaS;* adhesin	adhesin	Surface structures
5385	BB	2314		1245	0.47	conserved hypothetical protein		
5378	BB	[2321]		[1253]	0.05	intergenic region		
5377	BB	2322		1254	0.11	hypothetical protein		
7806	BB	2330	2620	1269	0.40	probable membrane efflux protein		Transport/binding proteins
5370	BB	2341		1277	0.48	probable AraC-family transcriptional regulator		
5369	BB	2342		1278	0.32	putative exported protein		
5368	BB	2343		1279	0.32	conserved hypothetical protein		
5367	BB	2344		1279	0.40	putative exported protein (BPP partial)		
5365	BB	2345		1280	0.37	putative exported protein (BPP partial)		
5362	BB	2347		1282	0.44	conserved hypothetical protein (BPP pseudogene)		
5360	BB	2348		1283	0.43	conserved hypothetical protein		
5361	BB	2349		1284	0.31	putative 2-pyrone-4,6-dicarboxylic acidhydrolase		
5358	BB	2350		1285	0.34	conserved hypothetical protein		
5355	BB	2352		1287	0.35	probable LysR-family transcription regulator (BPP pseudogene)		
5353	BB	2353		1288	0.36	conserved hypothetical protein		
5352	BB	2354		1289	0.47	putative exported protein		
3749	BP	2656	1868	1578	0.43	putative cytochrome (BP pseudogene)		Electron transport
7931	BB	2710		2775	0.40	Putative exported protein		
7932	BB	2711		2774	0.44	putative exported protein		
7933	BB	2712		2773	0.35	Putative sugar ABC transport system,substrate-binding protein		
7941	BB	2720		2765	0.24	Conserved hypothetical protein		
3521	BP	2732	1639	2754	0.42	putative exported protein (BPP pseudogene)		Inner membrane
7946	BB	2733	1638	2753	0.49	conserved hypothetical protein		Conserved in other organisms
650	BP	2735	1636	2751	0.40	putative nonspecific acid phosphatase precursor		Not classified (including putative assignments)
649	BP	2736	1635	2750	0.41	putative arylsulfatase protein		Other
664	BP	2742	1662	2744	0.14	putative DNA-binding protein		Not classified (including putative assignments)
3612	BP	2743	1663	2743	0.18	conserved hypothetical protein (BP partial)		Conserved in other organisms
5270	BB	2750			0.25	putative NADH:flavin oxidoreductase		Not classified (including putative assignments)
5267	BB	2752			0.39	LysR-family transcriptional regulator		Global regulatory functions
7971	BB	2839	1333	2669	0.49	hypothetical protein		Unknown function, no known homologs
636	BP	2942	1609	2976	0.18	probable aldehyde reductase		Not classified (including putative assignments)
634	BP	2943	1608	2977	0.18	putative membrane protein (BP pseudogene)		Conserved in other organisms
2686	BP	2944	1607	2978	0.22	probable LysR-family transcriptional regulator		Global regulatory functions
3621	BP	2963	1586	2997	0.12	conserved hypothetical protein		Conserved in other organisms
1211	BP	3061	2645	3098	0.50	putative IclR-family regulatory protein		Global regulatory functions
1209	BP	3063	2647	3100	0.21	putative exported protein		Periplasmic/exported/lipoproteins
1208	BP	3064	2648	3101	0.30	conserved hypothetical protein		Conserved in other organisms
3400	BP	3065	2649	3102	0.19	conserved hypothetical protein		Conserved in other organisms
8029	BB	3128		2807	0.38	putative membrane protein		
8030	BB	3130		2809	0.37	putative exported protein		
8037	BB	3160		2839	0.08	putative membrane protein		
8173	BB	multiple	1201	1617	0.49	*tcfA;* tracheal colonization factor precursor and other autotransporters	adhesin	Pathogenicity islands/determinants
6083	BPP	3291		1618	0.44	*tcfA;* tracheal colonization factor precursor	adhesin	
5392	BB	3424		1684	0.27	fimbrial protein	adhesin	
5408	BB	3479	3354		0.32	phage-related hypothetical protein	prophage	Unknown function, no known homologs
8058	BB	3484	3359		0.27	phage-related hypothetical protein		Phage-related functions and prophages
5567	BB	3516			0.43	phage-related hypothetical protein	prophage	Phage-related functions and prophages
8074	BB	3521			0.28	*cro;* phage regulatory protein		Phage-related functions and prophages
5105	BB	3538			0.11	phage-related conserved hypothetical protein		Phage-related functions and prophages
5106	BB	3539			0.35	phage-related hypothetical protein		Phage-related functions and prophages
5107	BB	3540	0504		0.12	phage-related hypothetical protein	prophage	Periplasmic/exported/lipoproteins
8081	BB	3542			0.22	putative phage excisionase		Phage-related functions and prophages
5108	BB	3543			0.37	phage integrase		Phage-related functions and prophages
5345	BB	3614			0.16	phage-related conserved hypothetical protein		Phage-related functions and prophages
5340	BB	3620			0.21	phage-related conserved hypothetical protein	prophage	Phage-related functions and prophages
5334	BB	3629			0.25	phage-related conserved hypothetical protein		Phage-related functions and prophages
443	BP	3893	2456	3443	0.47	*alcA;* alcaligin biosynthesis enzyme	iron uptake	Pathogenicity islands/determinants
439	BP	3897	2460	3447	0.25	*alcE;* putative iron-sulfur protein		Pathogenicity islands/determinants
188	BP	3978	3439	3543	0.21	*dnt;* dermonecrotic toxin	toxin	Colicin-related functions
8140	BB	4124		3690	0.50	putative enzyme-regulator fusion protein		
8141	BB	4125		3691	0.08	putative ring-hydroxylating dioxygenase large subunit		
8142	BB	4126		3692	0.04	putative ring-hydroxylating dioxygenase small subunit		
8143	BB	4127		3693	0.13	putative LysR-family transcriptional regulator		
8144	BB	4128		3694	0.11	putative exported protein		
8145	BB	4129		3695	0.07	putative hydrolase		
8146	BB	4130		3696	0.15	conserved hypothetical protein		
8147	BB	4131		3697	0.07	putative dioxygenase		
8148	BB	4132		3698	0.32	putative enzyme		
8149	BB	4133		3699	0.12	conserved hypothetical protein		
5546	BB	4328			0.37	*bbuR;* LysR-family transcriptional regulator		Global regulatory functions
5547	BB	4329			0.31	putative exported protein		Periplasmic/exported/lipoproteins
5550	BB	4331			0.42	putative exported protein		Periplasmic/exported/lipoproteins
3733	BP	4689	0612		0.16	putative exported protein		Periplasmic/exported/lipoproteins
2920	BP	4808	0672	4220	0.36	acetyl-CoA synthetase		Carbon compounds
3696	BP	4818	3731	4230	0.45	conserved hypothetical protein		Unknown function, no known homologs
6157	BPP	4887		4300	0.45	putative glutaredoxin		
2502	BP	4890	3783	4304	0.23	pertussis toxin subunit 1 precursor		Pathogenicity islands/determinants
3763	BP	4891	3784	4305	0.12	pertussis toxin subunit 2 precursor (BPP pseudogene)		Pathogenicity islands/determinants
3762	BP	4891	3784	4305	0.18	pertussis toxin subunit 2 precursor (BPP pseudogene)		Pathogenicity islands/determinants
2500	BP	4892	3785	4306	0.23	pertussis toxin subunit 4 precursor		Pathogenicity islands/determinants
2499	BP	4893	3786	4307	0.34	pertussis toxin subunit 5 precursor		Pathogenicity islands/determinants
3764	BP	4894	3787	4308	0.28	pertussis toxin subunit 3 precursor		Pathogenicity islands/determinants
3765	BP	4894	3787	4308	0.35	pertussis toxin subunit 3 precursor	pertussis toxin subunits and export apparatus	Pathogenicity islands/determinants
2498	BP	4895	3788	4309	0.37	pertussis toxin transport protein		Transport/binding proteins
2497	BP	4896	3789	4310	0.33	pertussis toxin transport protein		Transport/binding proteins
2496	BP	4897	3790	4311	0.23	putative bacterial secretion system protein		Transport/binding proteins
3317	BP	4898	3792	4312	0.24	putative bacterial secretion system protein		Transport/binding proteins
2495	BP	4899	3793	4313	0.33	putative bacterial secretion system protein		Transport/binding proteins
2493	BP	4901	3795	4315	0.35	putative bacterial secretion system protein		Transport/binding proteins
2492	BP	4902	3796	4316	0.47	putative bacterial secretion system protein		Transport/binding proteins
5000	BB	4986			0.40	putative exported protein		Periplasmic/exported/lipoproteins
1246	BP		2606		0.32	putative exported protein		Periplasmic/exported/lipoproteins
331	BP		3364		0.32	phage-related conserved hypothetical protein	prophage	Phage-related functions and prophages
323	BP		3374		0.16	phage-related hypothetical protein		Unknown function, no known homologs
1935	BP		0060	0524	0.41	conserved hypothetical protein		Conserved in other organisms
1934	BP		0061	0523	0.28	putative DNA-binding protein		Global regulatory functions
6027	BPP			0378	0.08	conserved hypothetical protein		Conserved in other organisms
6028	BPP			0379	0.35	conserved hypothetical protein		Conserved in other organisms
6029	BPP			0380	0.29	acetyltransferase (GNAT) family protein		Not classified (including putative assignments)
6044	BPP			0495	0.12			Phage-related functions and prophages

[a] Species from which the PCR product was amplified
[b] ORF numbers as annotated by the Sanger Centre sequencing team
[c] Fold difference between mean Cy5/Cy3 ratio in B. bronchiseptica complex IV versus complex I.
[d] Gene name and product as annotated by the Sanger Centre sequencing team

[e] Selected functional annotations
[f] ORF numbers between brackets indicate intergenic regions

Contiguous colors indicate that the probes are clustered on the genome.
