# Supplementary material for: Bordetella pertussis, the Causative Agent of Whooping Cough, Evolved from a Distinct, Human-Associated Lineage of B. bronchiseptica
Source: PLoS Pathog. 2005 Dec 30;1(4):e45. doi: 10.1371/journal.ppat.0010045 (PMC1323478; doi:10.1371/journal.ppat.0010045)
Supplement: Table S3 — (41 KB PDF) [file ppat.0010045.st003.pdf]

Table S3. Primer characteristics for the genes used in multilocus sequence typing, pertactin sequencing and in the detection of the insertion sequence elements

Gene name and product[a]	BB ORF[b]	BPP ORF[b]	BP ORF[b]	Primer Name	Sequence (5'→3')	Product length (bp)	Purpose	T_{ANN} (°C)
adk ; adenylate kinase	BB2005	BPP2560	BP2769	Adk-F	AGCCGCCTTTCTCACCCAACACT	513	A,S[d]	54
				Adk-R	TGGGCCCAGGACGAGTAGT		A,S	
fumC ; fumarate hydratase class II	BB4054	BPP3619	BP0248	FumC-F	CGTGAACCGGGGCCAGTCGTC	499	A,S	54
				FumC-R	GGCCAGCCAGCGCACATCGTT		A,S	
icd ; isocitrate dehydrogenase	BB3924	BPP3475	BP2488	Icd-F	CTGGTCCACAAGGGCAACAT	531	A,S	54
				Icd-R	ACACCTGGGTGGCGCCTTC		A,S	
glyA ; serine hydroxymethyltransferase	BB4348	BPP3875	BP2952	GlyA-F	CAACCAGGGCGTGTACATGGC	500	A,S	54
				GlyA-R	CCGCGATGACGTGCATCAG		A,S	
tyrB ; aromatic amino-acid aminotransferase	BB2272	BPP2024	BP1795	TyrB-F	CGAGACCTACGCTTATTACGAT	509	A,S	54
				TyrB-R	TGCCGGCCAGTTCATTTT		A,S	
pepA ; cytosol aminopeptidase	BB3736	BPP3285	BP2421	PepA-F	CGCCCCAGGTTGAAGAAAATCGTC	509	A,S	54
				PepA-R	ATCAGGCCCACCACATCCAG		A,S	
pgm ; phoshoglucomutase	BB0885	BPP0800	BP3141	Pgm-F	CGCCCATGTCACCAGCACCGA	545	A,S	54
				Pgm-R	CGCCGTCTATCGTAACCAG		A,S	
IS*481* ; transposase	-	-	multiple	IS481-F	GGGGTCACCGCGCCGACTGT	289	A	60
				IS481-R	GGGCCTGATGCTCGTAGCGC		A	
IS*1001* ; transposase	-	multiple	-	IS1001-F	CGCCGCTTGATGACCTTGATA	498	A	60
				IS1001-R	CACCGCCTACGAGTTGGAGAT		A	
IS*1002* ; transposase	-	multiple	multiple	IS1002-F	TCCCAGCTCCACGCACACCG	705	A	54
				IS1002-R	AACAACCATAAGCATGCGCG		A	
IS*1663* ; transposase	-	-	multiple	IS1663-F	GGGTCTGTATCACGAGCAAGCGG	265	A	60
				IS1663-R	CTTTGCGATTGAGCTCACGCAAC		A	
prn ; pertactin	BB1366	BPP1150	BP1054	Prn-SPF1	TCCCTGTTCCATCGCGGTG	1729, 1720, 1732[c]	A,S	60
				Prn-SPR3	GTTGGCGGCCAATCGATAGC		A,S	
				Prn-SPF2	ATCGCGCTCTATGTGGCCG	2399, 2426, 2389[c]	A,S	60
				Prn-SPR1	CCTGAGCCTGGAGACTGGCAC		A,S	
				Prn-SPF3	CACCGCACGGCAATGTCATC		S	
				Prn-SPF4	GGCGACCTTTACCCTTGCCAA		S	
				Prn-SPR2	CAGCGTCGCGTCCAGGTAGA		S	
				Prn-SPR4	GCAAGGTGATCGACAGGGGC		S	
				Prn-SPR5	TGGACCGTGACATTGGCGC		S	

[a] Gene name and product as annotated by the Sanger Centre sequencing team
[b] ORF numbers as annotated by the Sanger Centre sequencing team
[c] PCR fragment sizes in *B. bronchiseptica, B. parapertussis, B. pertussis,* respectively
[d] Abbreviations: A=amplification; S=sequencing
